# Single‐cell analysis of microglial transcriptomic diversity in subarachnoid haemorrhage

**DOI:** 10.1002/ctm2.783

**Published:** 2022-04-22

**Authors:** Junfan Chen, Lei Sun, Hao Lyu, Zhiyuan Zheng, Huasheng Lai, Yang Wang, Yujie Luo, Gang Lu, Wai Yee Chan, Sheng Guan, Yisen Zhang, Xinyi Chen, Zhongqi Li, Ho Ko, Kwok Chu George Wong

**Affiliations:** ^1^ Division of Neurosurgery, Department of Surgery, Faculty of Medicine The Chinese University of Hong Kong Hong Kong China; ^2^ Department of Interventional Neuroradiology The First Affiliated Hospital of Zhengzhou University Zhengzhou China; ^3^ Department of Neurology, Beijing Tiantan Hospital Capital Medical University Beijing China; ^4^ Department of Neurosurgery, The Second People's Hospital of Shenzhen First Affiliated Hospital of Shenzhen University Shenzhen China; ^5^ Department of Neurosurgery Hainan Branch of Chinese People's Liberation Army General Hospital Sanya China; ^6^ Department of Neurosurgery, Beijing Chaoyang Hospital Capital Medical University Beijing China; ^7^ CUHK‐SDU Joint Laboratory on Reproductive Genetics, Faculty of Medicine, School of Biomedical Sciences The Chinese University of Hong Kong Hong Kong China; ^8^ Department of Interventional Neuroradiology, Beijing Neurosurgical Institute, Beijing Tiantan Hospital Capital Medical University Beijing China; ^9^ Division of Neurology, Department of Medicine and Therapeutics, Faculty of Medicine Li Ka Shing Institute of Health Sciences, The Chinese University of Hong Kong Hong Kong China; ^10^ Department of International Medicine The Affiliated Hospital of Qingdao University Qingdao University Qingdao China; ^11^ Department of Neurology, Guangdong Neuroscience Institute Guangdong Provincial People's Hospital, Guangdong Academy of Medical Sciences Guangzhou China

Dear Editor,

Microglia‐mediated neuroinflammation plays a crucial role in subarachnoid haemorrhage (SAH).^1^, ^2^ However, the molecular characteristics of the activated microglia in SAH remain unclear. Here, we report the first detailed single‐cell transcriptomic characterisation of microglia post‐SAH, and we found specific biomarkers related to neuroinflammation in SAH.

In this work, the endovascular perforation (EVP) murine model^3^ was established with acceptable mortality, motor ability and weight change compared with the normal group and sham group (Figure S1). With immunohistochemistry, we noted prominent microglial cell accumulation in the cortex adjacent to the perforated site (CAPS) areas (Figure 1‐B and Figure S1). The accumulated microglia exhibited a more amoeboid‐shaped morphology, consistent with the activated states (Figures S2‐S4). Based on these results, we proceeded to perform single‐cell transcriptomic profiling of microglia and other brain cells on day 3 post‐SAH (Figure 1A; Excel S1‐S2). By using cell type‐specific marker gene expression patterns (Figure S5, *Tmem119* and *Cx3cr1* for microglia, others in **Method S9**), we classified single‐cell transcription into microglia (*n *= 5824) and other six cell types (*n* = 3,052) (Figure 1C,D). We termed these microglial clusters obtained from SAH models SAH microglial cluster (SMG‐C) (Figures S11‐S13). We further performed comparative bioinformatics of SAH (*n* = 5854) and healthy (*n* = 8160) mouse brain microglia and named it the integrated microglial cluster (IMG‐C) for supplementary analysis (Figures S23‐S26; Excel S3‐S4).

**FIGURE 1:**
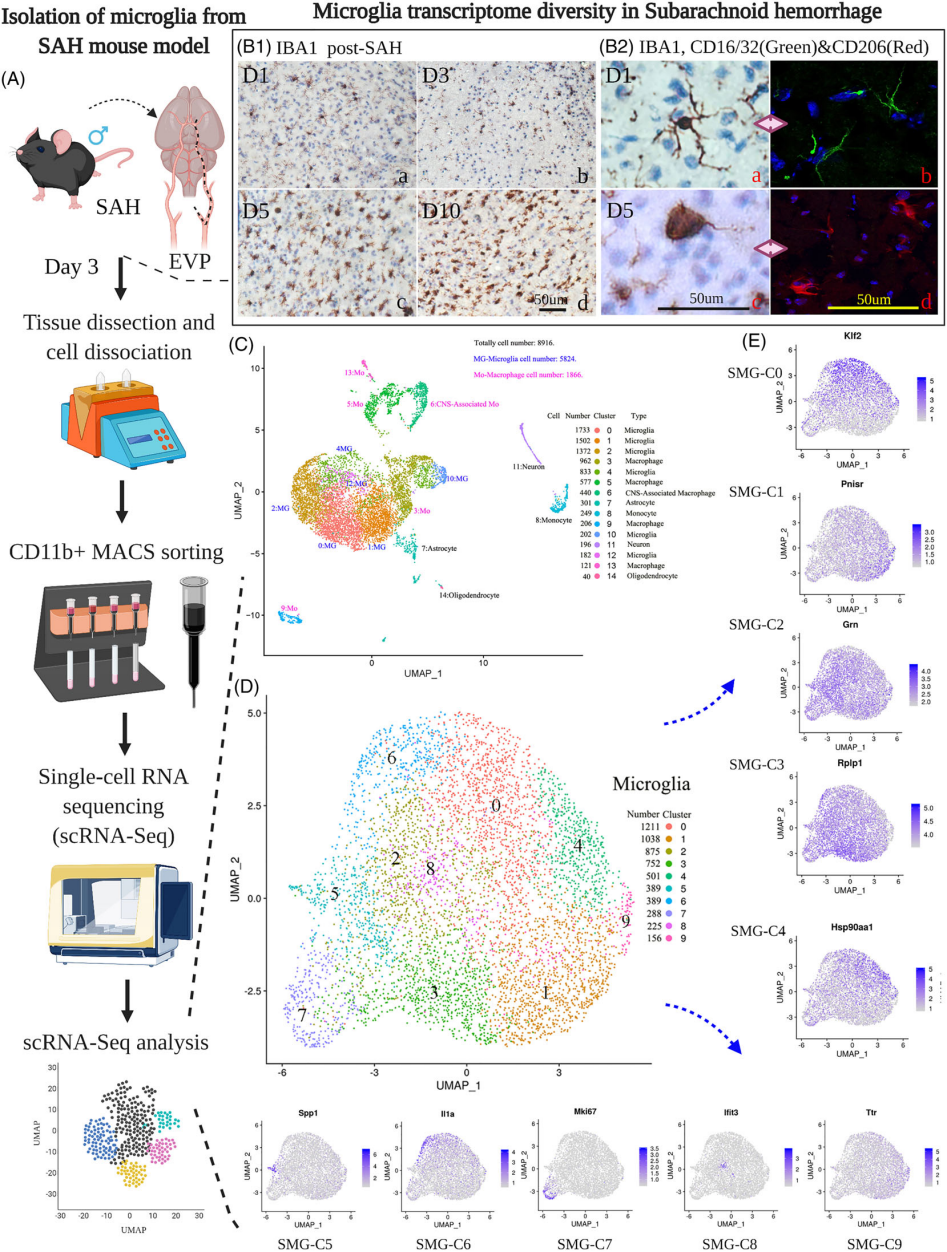
(A) Study design of single‐cell microglial transcriptomics in subarachnoid haemorrhage (SAH): the SAH model was induced and microglia were harvested from the mouse brain at day (D) 3 post‐SAH. The magnetic‐activated cell sorting (MACS) method was used to increase the throughput of CD11b‐positive cell isolation with high purity and viability for single‐cell RNA sequencing. (B1) Immunohistochemistry (IHC) staining of microglia on D1, D3, D5 and D10 showed that microglia accumulated at the cortex adjacent to the perforate site (CAPS) with time, increasing SAH. (B2) IHC staining of accumulated microglia showed a more amoeboid‐shaped morphology, consistent with the activated states. Immunofluorescence staining of CD16/32 (green) and CD206 (red) showed that M1‐like cells and M2‐like cells emerged on D1 and D5 after SAH. (C) Uniform manifold approximation and projection (UMAP) results showed microglia (5824) and central nervous system (CNS)‐associated macrophages (*n* = 440), macrophages (*n* = 1866), astrocytes (*n* = 301), monocytes (*n* = 249), neurons (*n* = 196) and oligodendrocytes (*n* = 40). (Markers can be found in the Supporting Information). (D) Upon visualisation with dimensionality reduction by UMAP, the microglia transcriptomes did not fall into distinct clusters but rather spanned a continuum of SAH microglia (SMG)‐10 clusters. We employed unsupervised clustering to classify the microglia into 10 distinct clusters, as this permitted us to examine the transcriptomic features of their different states and investigate the molecular diversity of microglia post‐SAH. (E): Top listed biomarkers of 10 clusters in SMGs. These clusters constituted 2.7%–20.8% of all microglia, and each was characterised by a panel of highly expressed genes

Among microglia in the SAH‐affected brain, three distinct subsets of SMG‐C5 (SAH‐associated microglia‐SAM), SMG‐C6 and SMG‐C7 were observed, whereby the molecular features of SMG‐C5 and SMG‐C6 differed from the previously reported activating microglial states^4^ (Figures S6, S7, S10, S11; Excel S2). SMG‐C5 was characterised by the expression of marker genes, including *Spp1, Lpl, Apoe, Ctsb, Lgals1, Lgals3, Fabp5, Mif, Lilrb4a, Cst7* and *Vim*. Other genes with high expression in SMG‐C5 included *Anxa2, Apoc2, CD63, CD72, Ctsc and Ctsz—*that have also been found to be upregulated in developing mouse brain microglia, Alzheimer's disease (AD) and lysolecithin (LPC) injury mouse models^4^, ^5^, ^6^ (Excel S2). According to prior reports, disease‐associated microglia (DAM) and injury‐responsive microglia (IRM) share the expression of a core set of genes encompassing *Spp1*, *Apoe* and *Lpl*,^5^ which are also upregulated in SAM. Meanwhile, important differences between the SAM and DAM transcriptomes suggest that microglia have distinct activation modes in SAH. For example, unlike DAM, SAM does not have elevated expression of *Trem2, Clec7a, Ccl6, or Axl*, but SAM has a unique panel of highly expressed genes, such as *Spp1, Lgals1, Lgals3, Fabp5, Mif, Lilrb4a* and *Vim* ^4^ (Excel S2). *Spp1*, *Lgals*, *Mif* and *Fabp5* are related to the enhancement of myelination, microglia regulation and activation.^7^ Gene Ontology (GO) and Kyoto Encyclopedia of Genes and Genomes (KEGG) analyses (Figures S7‐S9) further revealed that SMG‐C5 is closely related to oxidative phosphorylation, lysosomes and apoptosis (Figure S7). These findings suggest that SMG‐C5 is an important subgroup of the post‐SAH injury response.

SMG‐C6 expressed a variety of cytokines (*Il1a*, *Il1b, Tnf*, *Ccl4* and *Ccl3)*, chemokines (*Cxcl10* and *Cxcl2*), and other immune signal‐regulating genes (*CD83, CD74*, *CD14*, *Nfkbia* and *Nfkbiz*) (Figures S6, S7, S11, Excel S2). Some of these markers have been identified in previous studies on AD, multiple sclerosis (MS) and aging disease,^4^, ^5^, ^7^ overexpression of *Il1a, Ccl4* and *Ccl3* has been observed in the IRM subpopulation in the MS mouse model.^5^ These genes are related to the interleukin, tumour necrosis factor (TNF), toll‐like receptor 4 (TLR4) and nuclear factor‐kappa B (NF‐κB) signalling pathways in the GO results, which suggested that possible pathways were activated. Notably, some genes expressed in developing microglia were found to be upregulated in the post‐SAH state, including the proliferation genes *Birc5, Mki67* and *Fabp5* in SMG‐C7 and the metabolically active genes *Mif* and *Ms4a6c* in SMG‐C5.^8^ (Figures S7, S11 and Excel S2) The re‐expression of microglial developmental genes has also been found in previous studies, which may represent a special way microglia adopt to cope with stress and potentially play an important role in the activation of microglia and the pathogenesis of SAH.^5^ In the integration analysis, it was also found that the genes related to SMG‐C5 (IMG‐C6), SMG‐C6 (IMG‐C3) and SMG‐C7 (IMG‐C7) were highly expressed only in SAH microglia but not in normal microglia, thus further confirming the existence of these subgroups in SAH microglia (Figures S23, S25 and S26; Tables S1 and S2; Excel S3 and S4). By comparison with normal microglia belonging to the same IMG subgroup of SAH microglia, genes with strong expression changes in SMG‐C5, SMG‐C6 and SMG‐C7 were revealed, which mediate the key pathways driving the generation of specific post‐SAH microglial clusters (Tables S1 and S2). Trajectory analysis (SMG) also showed that the generation of SMG‐C5 is of profound significance to SMG‐C6 and SMG‐C7 (Figure S22). This evidence suggests that microglia have altered transcription in SAH, especially in these clusters.

We analysed the interaction of microglial subsets in the SAH state and found that microglia in SMG‐C5, SMG‐C6 and SMG‐C7 had the most frequent inflammatory pathway connection (Figures 2 and 3; Figures S14‐S16). The elevation of signalling pathways, including CCL, GALECTIN, TGFb, APP and SPP1, etc., was the greatest, which could affect the development and progression of neuroinflammation after SAH (Figure 2). While our results emphasised the importance of microglia in neuroinflammation (e.g., CCL, CD45, TGFb and Galectin), other cells, such as central nervous system (CNS)‐associated macrophages, astrocytes, neurons, monocytes and macrophages, could also be involved in neuroinflammatory responses after SAH (Figures S17‐S21; Figure S24). The interplay between brain cells should be taken into account in future SAH studies.^9^ (See Figure 4)

**FIGURE 2:**
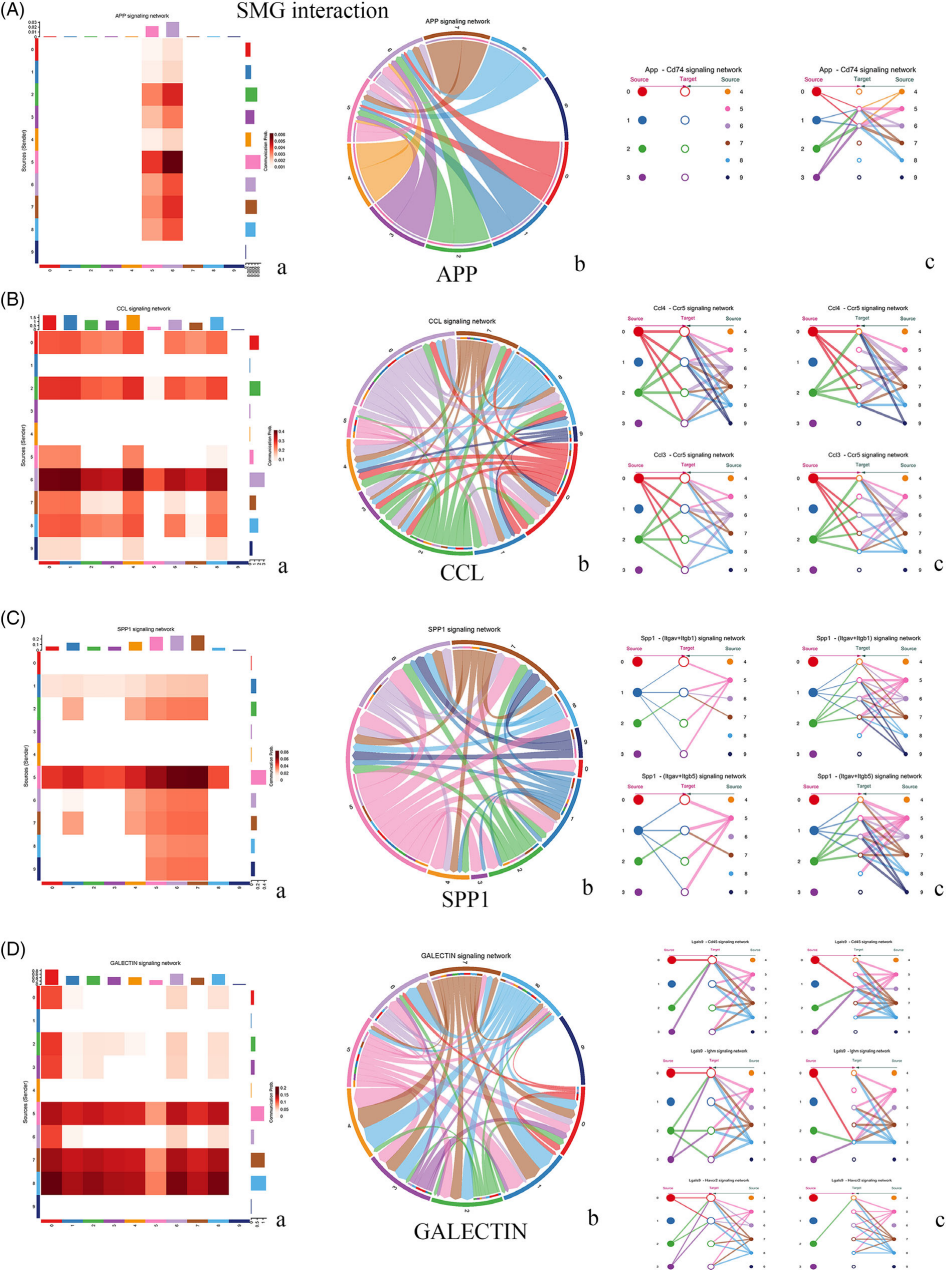
Some of the upregulated pathways in subarachnoid haemorrhage microglia (SMG) microglial subset interactions. (A) The amyloid‐β precursor protein (APP) signalling pathway was obviously upregulated in post‐SAH microglia. SMG‐C5 (SAM) and SMG‐C6 are the only targets of the APP signalling pathway. App – Cd74 is the main signalling network. (B) The CC‐motif chemokine ligand (CCL) signalling pathway was the most upregulated pathway after SAH. Almost all the microglial subsets joined this signalling pathway, mainly through Ccl4 and Ccl3 with the Ccr5 signalling network. (C) Secreted phosphoprotein 1 (SPP1). D: The GALECTIN signalling pathway. (a, heatmap; b, chord map and c, hierarchy connection)

**FIGURE 3:**
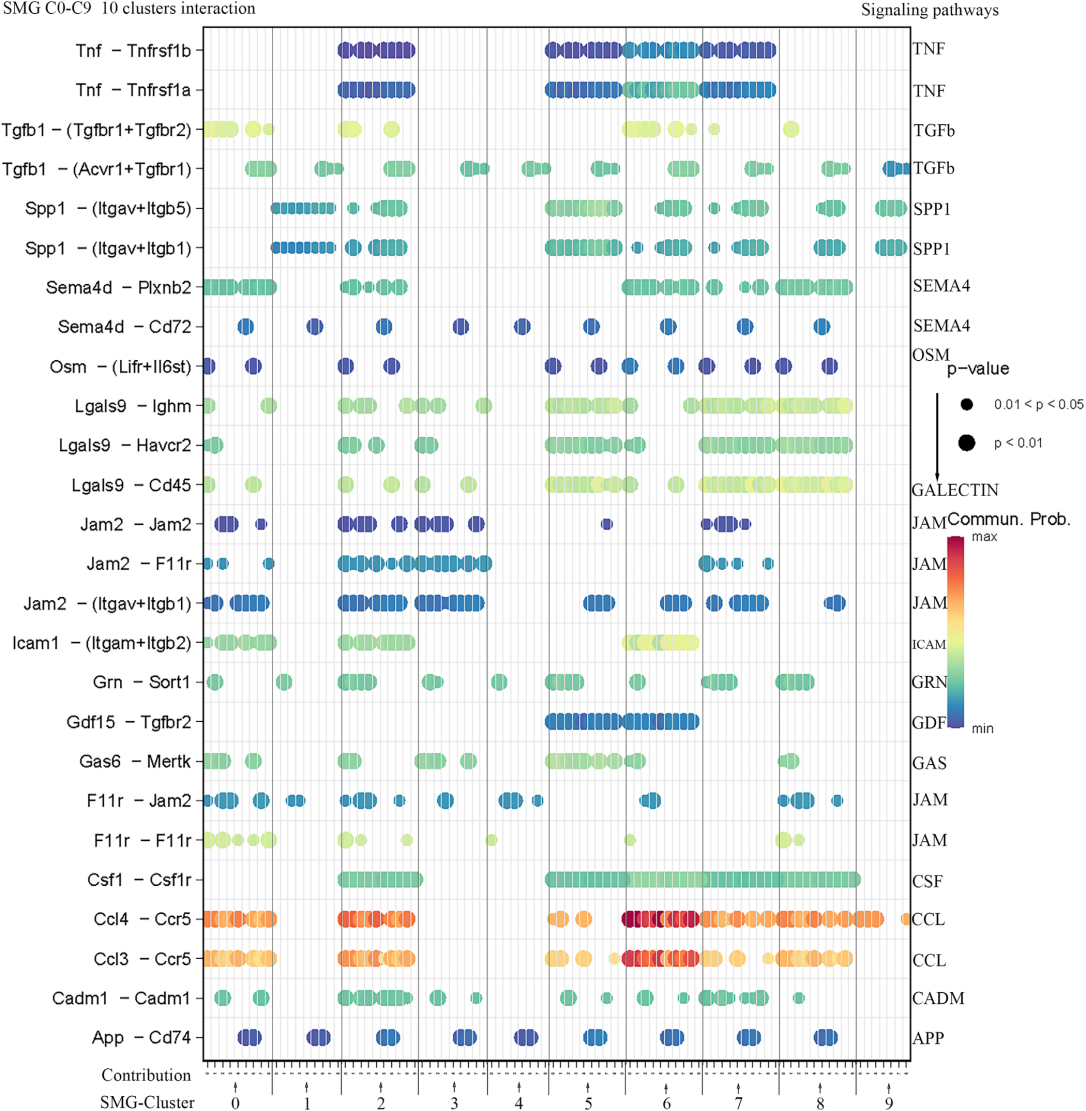
Subarachnoid haemorrhage microglia (SMG) microglia subset interaction. (In total, 15 signalling pathways.) Significantly upregulated signalling pathways found, according to the order of expression, were as follows: CC‐motif chemokine ligand (CCL), GALECTIN, transforming growth factor‐β (TGF‐β), junction adhesion molecule (JAM), intercellular adhesion molecule (ICAM), growth arrest‐specific (GAS), secreted phosphoprotein 1 (SPP1), progranulin (GRN), semaphorin 4 (SEMA4), cell adhesion molecule (CADM), colony‐stimulating factor (CSF), tumour necrosis factor (TNF), amyloid‐β precursor protein (APP), growth differentiation factor (GDF) and oncostatin M (OSM). The top five signalling roles are CCL, GALECTIN, TGF‐β, JAM and ICAM

**FIGURE 4:**
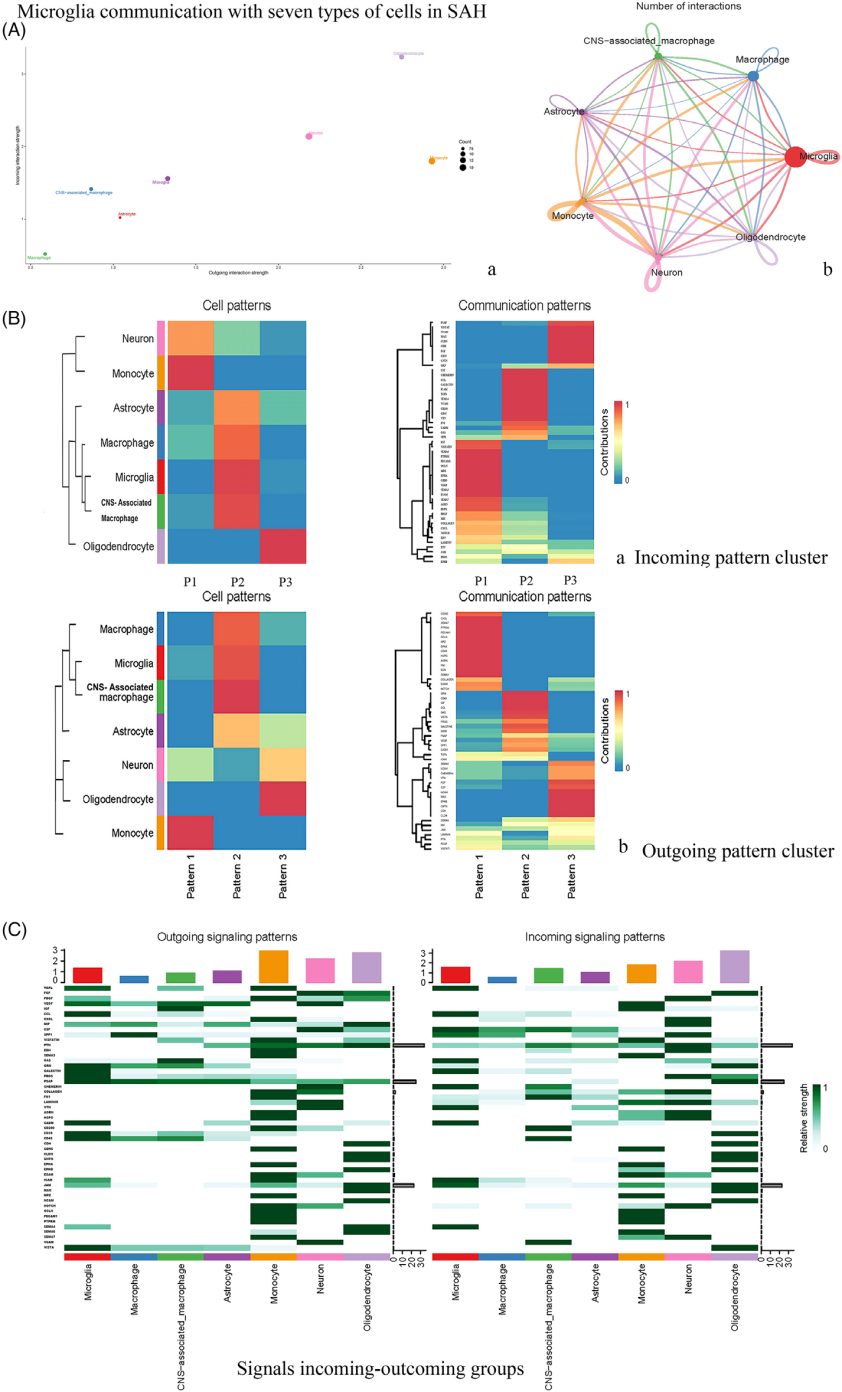
Microglia interactions with seven types of cells. (A) Microglia with the most communication with other kinds of cells and the communication is strong (a, cell communicating strength; b, cell interactions); (B) Microglia and central nervous system (CNS)‐associated macrophages with tight connections in cell communications, including colony‐stimulating factor (CSF), CHEMERIN, CC‐motif chemokine ligand (CCL), GALECTIN, transforming growth factor‐β (TGF‐β), intercellular adhesion molecule (ICAM), semaphorin 4 (SEMA4), vascular cell adhesion molecule (VCAM), CD200, CD45 and vitronectin (VTN) signalling pathways in the incoming pattern and progranulin (GRN), CD45, insulin‐like growth factor (IGF), CCL, gaseous (GAS) and V‐domain immunoglobulin suppressor of T‐cell activation (VISTA) in the outcoming (a, cell incoming signal patterns and b, cell outgoing signal patterns). (C) Signals incoming and outgoing groups, for most of the pathways such as TGF‐β, CCL, chemokine (C‐X‐C mif) ligand 1 (CXCL) and GALECTIN, microglia is the most important cell type after SAH. These signalling pathways were also found to be increased in microglial subset interactions

In summary, transcriptional analysis revealed diverse SAH‐specific microglial subgroups and found a functional relationship of elevated signalling pathways through receptor–ligand interactions with microglial subsets. Microglia and other cell crosstalk further substantiates the importance of microglia in driving post‐haemorrhage responses. However, the inclusion of only male mice was a limitation of our research. In addition, the microglial transcriptome at multiple time points after SAH and sham operation is needed in the future. Collectively, our research could be a starting point, provide data and theoretical support for future work and may potentially accelerate the development of microglial inflammation modulators of SAH.^10^

## AUTHOR CONTRIBUTIONS

Junfan CHEN and Kwok Chu George WONG designed the study. Junfan CHEN carried out most of the experiments, bioinformatics analysis, manuscript writing, revising and figure preparation. Lei SUN, Hao LYU, Huasheng LAI, Zhiyuan ZHENG performed the SAH model establishment and some of the immunostaining. Lei SUN, Sheng GUAN, Yisen ZHANG and Yang WANG assisted in bioinformatics analysis. Yujie LUO, Gang LU and Wai Yee CHAN contributed to the conceptualization of the study. Xinyi CHEN and Zhongqi LI helped with the microglial isolation steps. Junfan CHEN, Ho KO and Kwok Chu George WONG wrote the manuscript with input from all authors.

## CONFLICT OF INTERESTS

The authors declare no conflict of interests.

## Supporting information



Supplementary Information

Supplementary Information

Supplementary Information

Supplementary Information

Supplementary Information
