# Supplementary material for: Single‐cell analysis of microglial transcriptomic diversity in subarachnoid haemorrhage
Source: Clin Transl Med. 2022 Apr 22;12(4):e783. doi: 10.1002/ctm2.783 (PMC9029016; doi:10.1002/ctm2.783)
Supplement: Supplementary file 5 — Supplementary Information [file CTM2-12-e783-s001.pdf]

# Supplemental Materials:

**Title:** Single-cell analysis of microglial transcriptomic diversity in subarachnoid hemorrhage

**Authors:** Junfan CHEN,[1,2] Lei SUN,[3] Hao LYU,[1,4], Zhiyuan ZHENG,[1,5] Huasheng LAI[1], Yang WANG,[6] Yujie LUO,[1] Gang LU,[7] Wai Yee CHAN,[7] Sheng GUAN[2], Yisen ZHANG,[8] Xinyi Chen,[9,10] Zhongqi Li,[9,11] Ho KO,[9,*] and Kwok Chu George WONG[1,*]

Ho KO

ho.ko@cuhk.edu.hk

Kwok Chu George WONG*

georgewong@surgery.cuhk.edu.hk

## I: Supplemental Methods

## 1. Animals:

C57BL/6 (wild type) were obtained from the Laboratory Animal Services Centre of the Chinese University of Hong Kong (male, 12 weeks, average weight 25–30g). The mouse was placed in separate ventilation cages and exposed to food and water freely at 23°C and 50-60% humidity in a 12-hour/12-hour day and night cycle. All procedures involving animals and their care have been approved by the Ethics Committee of the Chinese University of Hong Kong.

## 2. SAH model perforate:

Prematurity subarachnoid hemorrhage (SAH) model was adopted in this study. [1,2] In simple: mice were anesthetized and fixed in a supine position. A surgical microscope was used in the whole process of model establishment. Firstly, a 1 cm incision was made in the midline of the mice neck, and then the left common carotid artery (CCA), left external carotid artery (ECA) and left internal carotid artery (ICA) were dissected clearly. ECA was ligated at the distal end, and two 1.5-cm-length 5–0 silk sutures were prepared for filament fixation. Block the blood of ECA and insert the filament (20-mm-long blunted 5–0 monofilament nylon suture) to ECA and ICA continue to the intracranial vessels. The vessel was perforated at the bifurcation of the middle cerebral artery (MCA) where the resistance was encountered. Then, the filament was immediately pulled out and then the hemorrhage was introduced into subarachnoid space. The sham model with the same procedure except for filament perforation. During the whole operation and recovery process, the mice were kept at 37 °C. To protect their eyesight, their eyes were coated with ointment. Buprenorphine was given intraperitoneally (i.p.) for analgesia twice a day for 3 days. On the day 1st, 3rd, 5th, and $10^{th}$ day after SAH induction, the body weight of mice was evaluated for wellbeing.

## 3. Phenotype evaluation:

Motor capacities was evaluated on the 1, 3, 5 and 10 days after SAH (nSHAM = nSAH = 6-8). To confirm SAH induction using two phenotypic tests: the holding time test and the Modified Bederson Score. The evaluating investigator was blinded to the

experimental conditions. The holding time test is adapted from the inverted grid test and has been widely used in SAH model assessment.[3,4] Briefly, a cotton-tipped applicator was placed and fixed on a pedestal at a 30 ° angle. Then, the mice were placed on it, and the time for the mice to remain in suspension was measured. Each mouse was measured three times to get the average time. The Modified Bederson score [5,6] was applied to evaluate neurological function. The mouse model of Moderate SAH model (Modified Bederson Score 2-3, **Table S4**) with the holding time test at D1 (range of 23.33±10.69) was recruited for the microglia study.

## 4. Immunohistochemistry:

Immunohistochemistry (IHC) was used to examine the condition of microglia. The paraffin brain sections (5 μm) were firstly through a xylene/ethanol dewax-rehydration series, then antigen retrieval was performed with citrate buffer for 20 minutes. Then, after the incubation of the first antibody and second antibody, endogenous peroxidize activity was quenched with 0.3% Hydrogen peroxide ($H_2O_2$). The brain slices were prepared in blocking buffer containing 2.5% goat serum, 1% Bovine serum albumin (BSA) for one hour and the primary antibody Iba1 (1:200; Abcam, #ab5076) was applied subsequently at 4 °C overnight. Envision+System-Horseradish peroxidase (HRP) secondary antibody was applied for 1 hour at room temperature. Finally, Diaminobenzidine (DAB) was utilized. Six random fields were examined on Cortex Adjacent to the Perforated Site (CAPS), Hippocampus (HIP) (The CA1 region of the hippocampus was selected for analysis), and Motor cortex (M1 cortex) (Left and right) respectively of each mouse under Microscope (Nikon) at 20X magnification. Microglial cell count was quantified by Image-Pro software.

## 5. Immunofluorescence:

Immunofluorescence (IF) was performed to define the post-SAH microglial polarization. Frozen sections were used for the IF, in simple, Mice were cardinally perfused with PBS followed by 10% buffered formalin then dehydration with the gradient concentration of sucrose solution from 15% to 30% and then embedded in the

Optimal cutting temperature (OCT) compound for cryosection. The frozen sections were immunolabeled with primary antibodies including CD16/32 (1:200; BD Biosciences, #553141), and CD206 (1:500; R&D, MMR, #AF2535), at 4 °C overnight. Fluorescence-conjugated secondary antibodies were then incubated with frozen sections, including Donkey anti - Rat Donkey DyLight 680 IgG H+L (1:200; Invitrogen, #SA5-10030), anti-Goat Alexa Fluor® 647 IgG H+L (1:200; Invitrogen, #A21447), and Donkey anti-Rat Alexa Fluor® 488 IgG H+L (1:200; Invitrogen, #A21208), at room temperature for 2 hours. Then washed with PBS and mounted with 4′,6-diamidino-2-phenylindole (DAPI) (Abcam, #ab104139). Immunofluorescent images were acquired using a microscope (Nikon Eclipse Ti Inverted Microscope, Nikon). Quantification of M1/M2 microglial phenotype was carried out in three randomly selected high power microscopic fields across three sections.

**6. Acute microglial isolation and purification:**

Centrifuges and tools are prechilled to 4°C or on ice. Mice (3-5 mice usually, for this study was four) at 3$^{rd}$-day post-SAH was anesthetized and then transcardially perfused with cold PBS for 2–4 minutes each mice using a 30 ml syringe with a 20 Gauge needle. Then quickly dissect the brains, put them in cold PBS, and wash them twice to remove the blood, hair and fiber. The olfactory bulb and the cerebellum were removed. Neural Tissue Dissociation Kit P was used for brain digestion. Cut brain into small pieces with sterile scissors, and put it into a gentleMACS™ C tube containing prewarmed enzyme solution for mechanical dissociation. Then, the C tube was inverted on the gentleMACS™ Dissociator, the program (Mouse brain program) was run, and reagents were added according to the commercial protocol (Miltenyibiotec) and published paper. [7-9] Pass cell digest over a 70 µm filter (Pre-wetted with 1ml PBS/BSA 0.5%) to remove cell clumps, then set into a 50 ml conical tube. Spin at 400 × g(RCF) and 4°C for 10 minutes and aspirate supernatant. Added adequate CD11b (Microglia) MicroBeads based on the cell number and incubated 15 minutes at 4°C. LS column and QuadroMACS were used for the positive selection of microglia. (1 LS column: Max.

number of labeled cells: $2\times10^7$) After positive selection, take out the LS column from the magnetic holder then push the solution through the LS column with the plunger provided. This will apply gentle pressure to remove the microglia from the LS column and to obtain Microglia Fraction. Centrifuge the Microglia Fraction at 300g for 10 minutes at 4°C and discard the supernatant. Put cell in 1.5ml EP tube also with 10ml 0.5% BSA-PBS buffer with 2μL RNAse inhabitor at 4 $^0$C. [7-10]

**7. Flow Cytometry:**

Microglia suspension was washed with 0.5% BSA-PBS and followed with cell surface staining at 4 °C for 30min using the following markers: Tmem119 Monoclonal Antibody-Alexa Fluor 488 (Invitrogen, #53-6119-80), CD11b-APC (Miltenyi Biotec, #130-113-793), Propidium Iodide Solution (Miltenyi Biotec, #130-093-233). Then cell will be analyzed on BD LSRFortessa™ Cell Analyzer (BD Biosciences) according to the manufacturer's instructions. Cell viability was assessed using the Trypan blue (Abcam, #Ab233465) cell analysis on a hemocytometer.

**8. Single cell RNA sequencing:**

CD11b positive cell suspension purified from MACS were sequenced by the Chromium single-cell gene expression platform (10x Genomics). According to the manufacturer's instructions, about 10,000-15,000 microglia of each sample were directly loaded into each sample and then combined into droplets with barcoded beads by using the Chromium controller and Chromium Single-Cell 3′ Reagent Kits v3 (10x Genomics). According to the manufacturer's specifications, the barcode library was generated, and then the samples were sequenced to an average depth of 40,000-60,000 reads on a DNBSEQ-PE100 (BGI).

**9. Single-cell data analysis and Bioinformatics:**

Sequencing results were demultiplexed and converted to FASTQ format using Illumina bcl2fastq software. Sample demultiplexing, barcode processing and single-cell 3' gene counting by using the Cell Ranger pipeline(https://support.10xgenomics.com/single-cell-geneexpression/ software/pipelines/latest/what-is-cell-ranger, version 3.1.0) and

scRNA-seq data were aligned to Ensembl genome GRCm38 reference genome, a total of 13,194 single cell captured from 4 SAH mouse brain were processed using 10X Genomics Chromium Single Cell 3’ Solution. The Cell Ranger output was loaded into Seurat (version 3.1.1) to be used for Dimensional reduction, clustering, and analysis of scRNA-seq data. Overall, 8,916 cells passed the quality control threshold: all genes expressed in less than 1 cell were removed, the number of genes expressed per cell was > 500 as low and <4000 as high cut- off, UMI counts less than 500, the percent of mitochondrial-DNA derived gene-expression <10%. By using Tmem119 and Cx3cr1[11] as confirmed microglia markers [7,12] (Cell marker: CNS- associated macrophage (*Mrc1, Lyve1*), oligodendrocyte (*Mobp. Mog*), astrocyte (*Rfx4, Gfap*), neuron (*Bcar3, Map2, 2010300C02Rik*), monocyte (*Ly6c1, Ly6c2*) and macrophage (*S100a6, Tgfbi, Itga4, Basp1*).), 5824 microglia were involved in bioinformatics. Previous biomarkers of other brain cell types were used.[13] Further, we recruit microglia transcriptome from normal adult mouse brain ScRNA-seq dataset (Microglia isolated by CD11b magnetic beads, and sequenced by 10X genomes platform (Same with our isolation and sequencing method), 4 normal male mice samples, age 14 weeks, C57BL/6, whole brain) and make integration analysis of SAH microglia (microglia number: 5854) and normal microglia (microglia number: 8160) with the same method with SAH microglia bioinformatics. For data from different experiments, Seurat CCA integration functions were used. [7,14] (Normal sample: GSM3442026, GSM3442027, GSM3442030 and GSM3442031)

To visualize the data, we further reduced the dimensionality of all 8,916 cells (For integration analysis were 14014 cells) using Seurat and used Uniform Manifold Approximation and Projection (UMAP) to project the cells into 2D space, The steps includes:1. Using the LogNormalize method of the "Normalization" function of the Seurat software to calculate the expression value of genes; 2. PCA (Principal component analysis) analysis was performed using the normalized expression value, Within all the PCs, the top 10 PCs were used to do clustering and UMAP analysis； 3. To find clusters, select the weighted Shared Nearest Neighbor (SNN) graph-based clustering method. Marker genes for each cluster were identified with the "bimod"

（Likelihood-ratio test）with default parameters via the FindAllMarkers function in Seurat. This selects markers genes which are expressed in more than 10% of the cells in a cluster and average log2 (fold change) of greater than 0.26. 4. Monocle2 was applied for the trajectory state analyzed and Cellchat was applied to analyze cell interaction. 5. Integration analysis with the same method above.

**10. Statistics:**

All the data were expressed as mean ± SEM. Statistical analyses were conducted by IBM SPSS 22.0 software. For the cross-sectional evaluation cohort, One-way ANOVA was used for statistical analysis to evaluate the microglia change across 4 time points. The independent t-test was used to determine the significance comparison between groups. The equality of error variance was tested as appropriate. $P<0.05$ after Bonferroni adjustment for multiple comparisons was considered statistically significant.

## Reference:


1. Zheng ZV, Lyu H, Lam SYE, Lam PK, Poon WS, Wong GKC. The Dynamics of Microglial Polarization Reveal the Resident Neuroinflammatory Responses After Subarachnoid Hemorrhage. *Translational stroke research*. Jun 2020;11(3):433-449. doi:10.1007/s12975-019-00728-5
2. Du G, Lu G, Zheng Z, Poon W, Wong K. Endovascular Perforation Murine Model of Subarachnoid Hemorrhage. *Acta neurochirurgica Supplement*. 2016;121:83-8. doi:10.1007/978-3-319-18497-5_14
3. Aartsma-Rus A, van Putten M. Assessing functional performance in the mdx mouse model. *Journal of visualized experiments : JoVE*. 2014;(85)doi:10.3791/51303
4. Gris T, Laplante P, Thebault P, et al. Innate immunity activation in the early brain injury period following subarachnoid hemorrhage. *Journal of neuroinflammation*. 2019;16(1):253. doi:10.1186/s12974-019-1629-7
5. Ruan J, Yao Y. Behavioral tests in rodent models of stroke. *Brain hemorrhages*. 2020;1(4):171-184. doi:10.1016/j.hest.2020.09.001
6. Lyu C, Zhang Y, Gu M, et al. IRAK-M Deficiency Exacerbates Ischemic Neurovascular Injuries in Experimental Stroke Mice. *Frontiers in cellular neuroscience*. 2018;12:504. doi:10.3389/fncel.2018.00504
7. Hammond TR, Dufort C, Dissing-Olesen L, et al. Single-Cell RNA Sequencing of Microglia throughout the Mouse Lifespan and in the Injured Brain Reveals Complex Cell-State Changes. *Immunity*. Jan 15 2019;50(1):253-271.e6. doi:10.1016/j.immuni.2018.11.004
8. Holt L, Stoyanof S, Olsen M. Magnetic Cell Sorting for In Vivo and In Vitro Astrocyte, Neuron, and Microglia Analysis. *Current protocols in neuroscience*. 2019;88(1):e71. doi:10.1002/cpns.71
9. Hickman S, El Khoury J. Analysis of the Microglial Sensome. *Methods in molecular biology*

*(Clifton, NJ)*. 2019;2034:305-323. doi:10.1007/978-1-4939-9658-2_23
10. Sharma K, Schmitt S, Bergner C, et al. Cell type- and brain region-resolved mouse brain proteome. *Nature neuroscience*. 2015;18(12):1819-31. doi:10.1038/nn.4160
11. Li Q, Cheng Z, Zhou L, et al. Developmental Heterogeneity of Microglia and Brain Myeloid Cells Revealed by Deep Single-Cell RNA Sequencing. *Neuron*. Jan 16 2019;101(2):207-223.e10. doi:10.1016/j.neuron.2018.12.006
12. Masuda T, Sankowski R, Staszewski O, Prinz M. Microglia Heterogeneity in the Single-Cell Era. *Cell reports*. Feb 4 2020;30(5):1271-1281. doi:10.1016/j.celrep.2020.01.010
13. Ochocka N, Segit P, Walentynowicz K, et al. Single-cell RNA sequencing reveals functional heterogeneity of glioma-associated brain macrophages. *Nature communications*. 2021;12(1):1151. doi:10.1038/s41467-021-21407-w
14. Satija R, Farrell JA, Gennert D, Schier AF, Regev A. Spatial reconstruction of single-cell gene expression data. *Nature biotechnology*. 2015/05/01 2015;33(5):495-502. doi:10.1038/nbt.3192

## II: Supplemental Tables (4)

**Table S1: Gene comparison between IMG and SMG clusters.**

| SMG clusters | IMG clusters |
|---|---|
| SMG-C5: SAM – (Spp1, Lpl, Apoe, Ctsb, Cst7 and Mif etc. ) | IMG-C6: 74.0% (77/104) genes crossed with SMG-C5. |
| SMG-C6: IAM - (Il1b, Il1a, Ccl4, Ccl3 and chemokines modulation genes: Cxcl10 and Cxcl2 etc. ) | IMG-C3: 80.3% (49/61) genes crossed with SMG-C6; |
| SMG-C7: PAM- (Birc5, Ccnb2, Cenpa and Mki67 etc.) | IMG-C7: 79.5% (70/88) genes crossed with SMG-C7. |
| SMG-C0: immediate early genes (Jun, Jund and Klf etc.) | IMG-C4: 75.0% (21/28) genes crossed with SMG-C0;<br>IMG-C3: 50.8% (31/61) genes crossed with SMG-C0. |
| SMG-C8: Interferon regulatory genes, including Ifit3, Ifitm3, Ifi204 and Ifi27l2a etc. | IMG-C9: 44.5% (57/128) genes crossed with SMG-C8. |
| SMG-C2: phagocytic capacity and injury related genes (Ctsb, Ctsd, Ccl3, Ccl4, Lgals3bp and Cst7 etc.) | IMG-C6: 9.6% (10/104) genes crossed with SMG-C2, but 10 is a half of SMG-C2 expression features. |
| SMG-C3: ribosome genes (Rplp1, Rplp0 and Rpl41etc.) | IMG-C1: 77.3% (34/44) crossed with SMG-C3. |
| SMG-C9: Ttr, Mbp, Mobp and Dennd1c et c. | IMG-C8: Mbp, Mrc1, Dalrd3 and Abca1 etc. |
| SMG-C1: homeostatic genes (P2ry12, P2ry13 and Siglech etc.) | IMG-C0: Sdf2l1 and Crybb1 etc.<br>IMG-C2: homeostatic microglia genes. |
| SMG-C4: Mafb, btg2 and Txnip etc. | IMG-C5: signaling inhibitors: Bmp2k, transcriptional repressors: Bhlhe41, Ncoa3, and Notch2 etc. |

Inflammatory-associated microglia: IAM; Integrated microglia: IMG; SAH microglia: SMG; SAH-associated microglia: SAM; Proliferation-associated microglia: PAM.

1

**Table S2: Different gene expression of SMG and normal microglia in IMG clusters.**

| Cluster | Genes silenced in SAH microglia | Top10 genes Upregulated in SAH microglia[a] | Top10 genes downregulated In SAH microglia[a] | Top 3 different genes | Enriched signal pathways (Genes, p value) |
|---|---|---|---|---|---|
| **IMG-C3 (SMG-C6)** | *Gm10116, Toporsos, Shfm1, Fam46a, Rpl13a.ps1, Usmg5, RP23.81F19.2, X2810428I15Rik, Zfos1, Gm8186, Gm8730 etc.* | *Klf2, Egr1, Atf3, Fos,* ***Cxcl2, Cxcl10****, Hspa1a, Hes1,Sepine1 and Dusp1* | ***Galnt7****, Cd63, CD83, Ccl2, Trim12c, Ccl5 Pum3, Hcar2 Ppp2r3c, Desi1* | ***Cxcl10, Cxcl2, Galnt7*** | GO: response to stress (115, 2.74E-16), immune system process (89, 1.48E-13), and positive regulation of macromolecule metabolic process (101, 5.44E-14), regulation of cell death (65, 2.64E-12). KEGG: CP: Apoptosis (14), Lysosome (10), EIP: MAPK (16), TNF (13) and NF-KB (8) signal pathway MB: Il-17(12) and NOD-like receptor signaling pathway (12); GIP: Ribosome. p<0.01. |
| **IMG-C4 (SMG-C0)** | *Same with IMG-C3* | ***Gadd45g, Zfp36, Adamts1****,Smad7, Hspa1a , Serpine1, Hes1 ,Csf1, Nfkbiz, Gm13889* | *Mrpl33, Wdr44, Foxp1, Nsrp1, Wdr89, Gm43603, Nsrp1, Tubb2a, Marf1, CD63* | ***Gadd45g ,Adamts1 , Zfp36*** | GO: negative regulation of biological process (140,1.56E-17) and translation (43, 9.50E-17) through mRNA binding (16, 5.10E-05) and RNA binding (11, 2.04E-07). KEGG: CP: Apoptosis (9), Gap junction (7). EIP: TNF (10), NF-KB (5), Apelin (9) and HIF-1(8) signal pathway and cytokine-cytokine receptor interaction (14). GIP: Ribosome. p<0.01. |
| **IMG-C6 (SMG-C5)** | *Same with IMG-C3* | ***Cxcl2, Cxcl10, Il1b,*** *Tnf, Ifi27l2a, Hmox1, Spp1, Serpine1, Hspa1a, Zfp36.* | *Cd63, Cd9, Serpinf1, Il4i1, Cd27, D430042O09Rik.* | ***Cxcl2, Il1b, Cxcl10*** | GO: defense response (42, 311E-17), immune system process (53, 1.79E-17), response to external stimulus (49, 9.15E-16), regulation of programmed cell death (38, 1.88E-17) and negative regulation of cell death (32, 3.35E-17) KEGG: EIP: TNF (10) and MAPK (9) signaling pathway OS: Osteoclast differentiation (8), IL-17 (9) and Toll-like receptor signaling pathway (7). GIP: |

| | | | | | |
|---|---|---|---|---|---|
| | | | | | Ribosome. P<0.01. |
| **IMG-C7 (SMG-C7)** | *Same with IMG-C3* | ***Cxcl2, Il1b,*** *Cxcl10, Spp1, Fabp5, Cd83, Serpine1, Gm13889, Klf4, Hspa1a* | ***Hells****, Mcm5, Dut, Gmnn, Lig, Csrp1, Stat5b, Slc16a10* | ***Hells, Cxcl2, Il1b*** | GO: regulation of cellular metabolic process (128, 2.58E-15), cellular nitrogen compounds metabolic process (132, 5.31E-16), cell cycle (70, 3.55E-18), cell death (72, 4.58E-15), metabolic (195, 5.17E-17) and response to stress (64, 1.86E-16).<br>KEGG: CP: Cell cycle (12), Apoptosis (10).<br>EIP: NF-KB (9), TNF (9) and MAPK (14) signaling pathway.<br>OS: Toll- like receptor (9) and IL-17(8) signaling pathway.<br>GIP: Ribosome and DNA replication. P<0.01. |
| **IMG-C9 (SMG-C8)** | *Same with IMG-C3* | ***Cxcl10,*** *Gadd45b, Hes1,* ***Serpine1****, Gem, Hspa1a* | *Ccl5, Sde2, Zfp131, Rpl39, Rpl37, Rps18, Isg20, Fundc1, Ccdc88b,* **Anks,** *CD63* | ***Cxcl10, Anks, Serpine1*** | GO: response to stress (93, 6.05E-17), Translation (37, 9.61E-16), peptide biosynthetic process (37, 2.05E-15).<br>KEGG: CP: Apoptosis (9),<br>EIP: TNF signaling pathway (9) and cytokine- cytokine receptor interaction (12).<br>OS: TLR (8), IL-17 (7), Chemokine (10), NOD-like receptor (10) and C-type lectin receptor signaling pathway (7). GIP: Ribosome. P<0.01. |

CP: Cellular process; EIP: Environmental information processing; GIP: Genetic information processing; Integrated microglia: IMG; SAH microglia: SMG; Gene ontology: GO; Kyoto Encyclopedia of Genes and Genomes: KEGG; Organismal systems: OS. [a] The absolute value of avg_logFC ≥0.26, p<0.05.

## Table S3: Key resources

| Reagent | Manufacturer | Cat. number |
|---|---|---|
| Anti- CD16/32 | BD Biosciences | 553141 |
| Anti- CD206 | R&D,MMR | AF2535 |
| Tmem119 Monoclonal Antibody, Alexa Fluor 488 | Invitrogen | 53-6119-80 |
| ART 1000G genomic racked ster | Thermo Fisher Scientifi | 2019G |
| Bovine Serum Albumin | Sigma | A9418-50G |
| CD11b-APC | Miltenyi Biotec | 130-113-793 |
| CD11b MicroBeads, mouse | Miltenyi Biotec | 130-049-601 |
| Cell strainers 70um | Falcon | 352350 |
| DNase I, grade II | Roche | 10104159001 |
| Gentle MACS 25 C Tubes | Miltenyi Biotec | 130-090-237 |
| Goat serum | Abcam | ab7481 |
| IBA1 | Abcam | Ab5076 |
| LS Separation columns | Miltenyi Biotec | 130-042-401 |
| Neural Tissue Dissociation Kit-Papain | Miltenyi Biotec | 130-092-628 |
| PBS | Thermo Fisher Scientifi | 10010031 |
| Propidium Iodide Solution | Miltenyi Biotec | 130-093-233 |
| RNASin(R) plus RNAse inhibitor | Promega-gm | N2615 |
| RNAseZAP | Thermo Fisher Scientific | AM9780 |
| Trypan Blue | Abcam | Ab233465 |
| **Equipment** | **Manufacturer** | **Cat. number** |
| QuadroMACS Separator | Miltenyi Biotec | 130-090-976 |
| MACS Multistand | Miltenyi Biotec | 130-042-303 |
| gentleMACS Dissociator | Miltenyi Biotec | 130-093-235 |
| BD LSRFortessa™ Cell Analyzer | BD Biosciences | |
| Nikon Eclipse Ti Inverted Microscope | Nikon | |

## Table S4: Modified Bederson Score

| Modified Bederson Score | Description |
|---|---|
| 4 | Longitudinal spinning or seizure activity. |
| 3 | Unidirectional circling |
| 2 | As for 1, plus decreased resistance to lateral push. |
| 1 | Forelimb flexion. |
| 0 | No deficit. |

## III: Supplemental Figures (26)

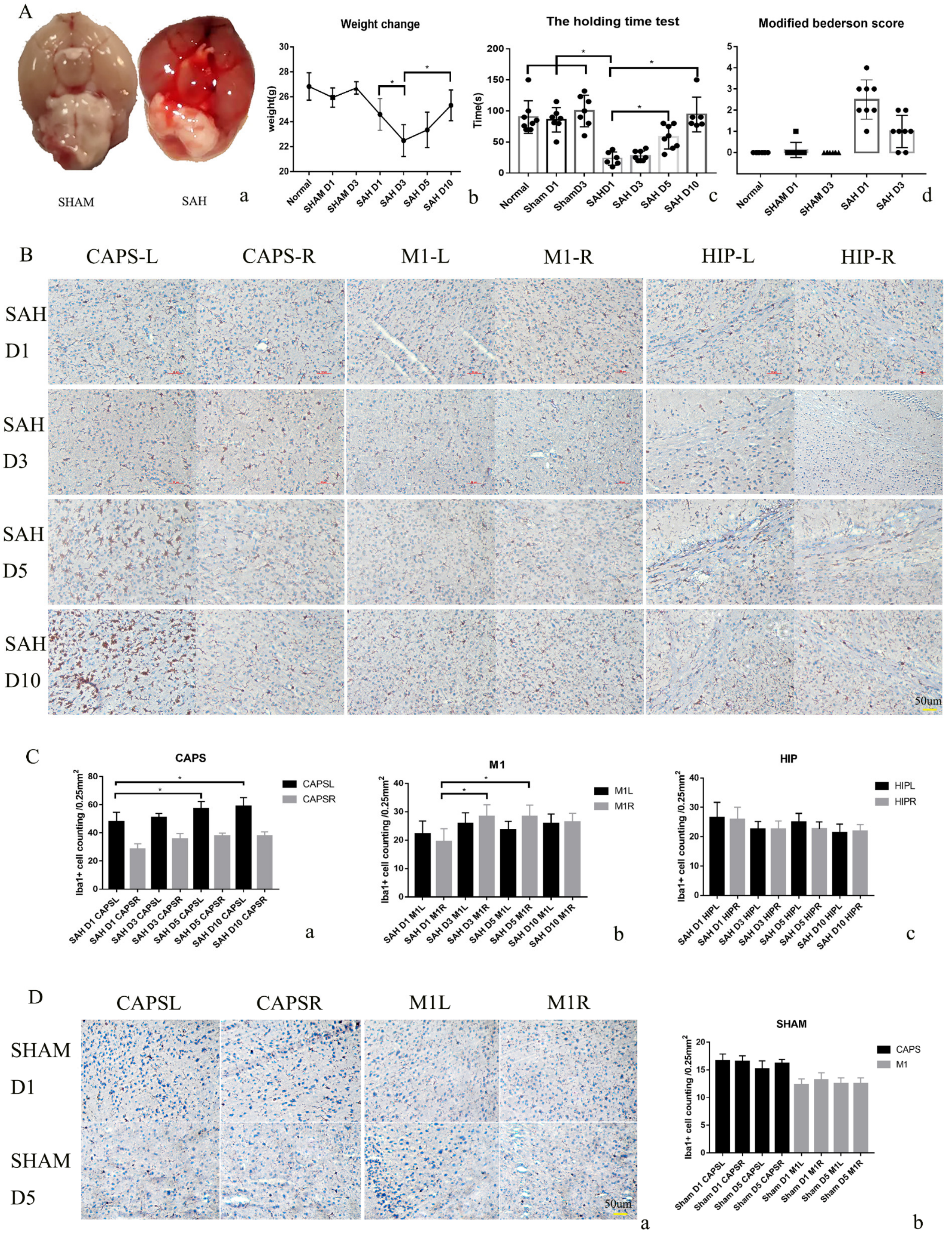


**Figure S1- A**: SAH model evaluation; a-b: On the third day after the endovascular perforation (EVP) procedure, the SAH group had the greatest weight loss and motor ability change on the holding time test, while the normal and sham control groups did not exhibit changes in these measures; c: Modified bederson score was used to fast evaluate SAH model, which shows obvious change in the day (D) 3 of mouse model. **B**: Iba1+ IHC staining in D1, D3, D5 and D10 of SAH. (Cortex Adjacent to the Perforated Site (CAPS), Hippocampus (HIP) (The CA1 region of the hippocampus was

selected for analysis), and Motor cortex (M1)), the morphology of microglia has obvious change with time increase, tend to be from ramified to amoeboid shape. **C**: Quantitative analysis of Iba1+ cell showed that microglia accumulated obviously in the CAPS areas after SAH with time increase and gradually stable in D10 which is consistent with microglia activation in SAH; **D**: Iba1+ IHC staining in D1 and D5 in sham group didn't see any obviously change. n=6-8, *$p<0.05$.

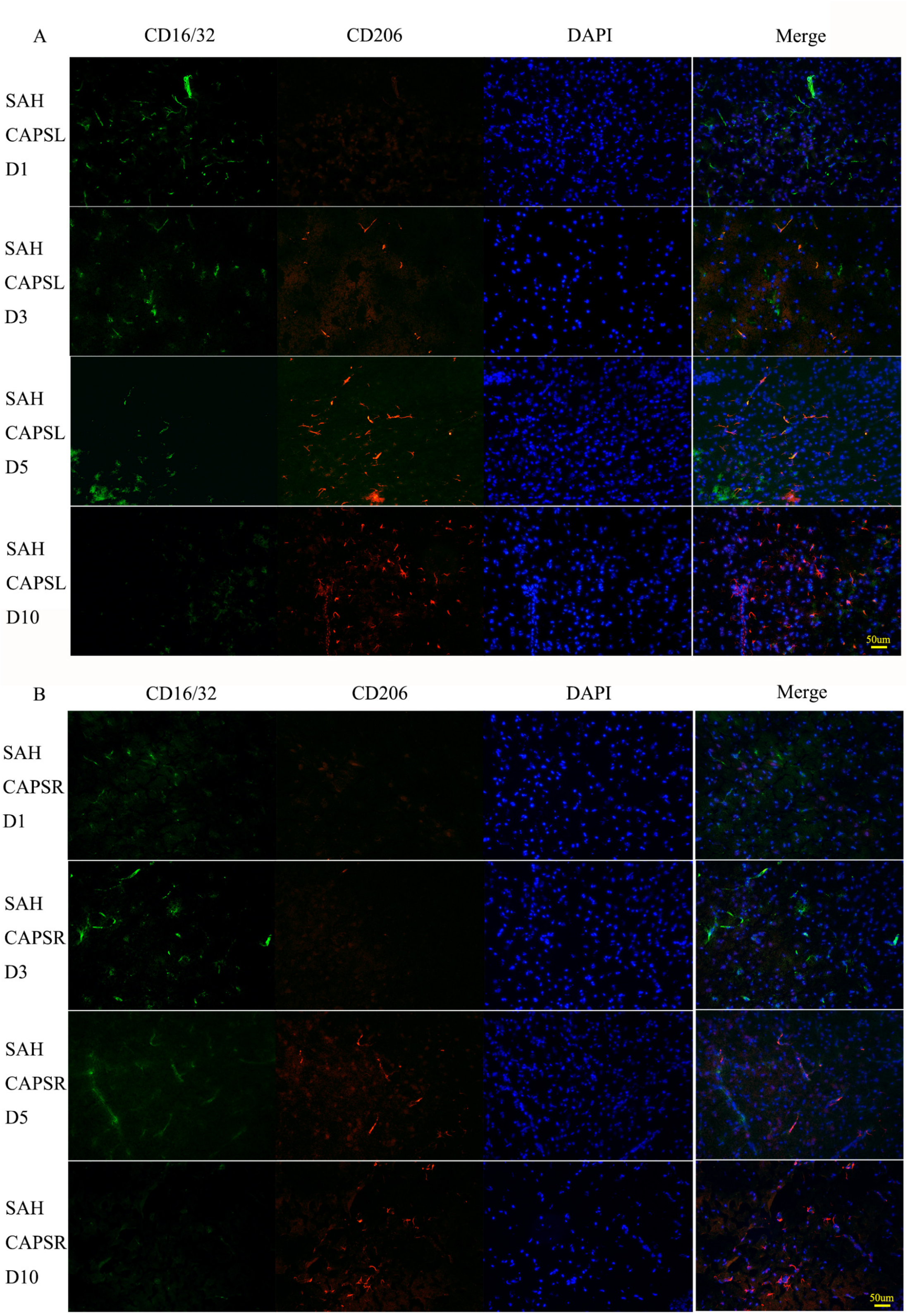


**Figure S2-** IF staining of CD16/32 (Green) and CD206 (Red), in D1, D3, D5 and D10 of SAH group in CAPS left (A) and CAPS right (B). CD16/32 was used to show proinflammation state and CD206 was used to show M2 like cells in the brain. After SAH, microglia are probably activated and transform their state with time change from

traditional proinflammation state (M1) to anti-inflammation state (M2) in the mice brain, especially different in Day 1 and Day 5, thus point the important stage of Day 3.

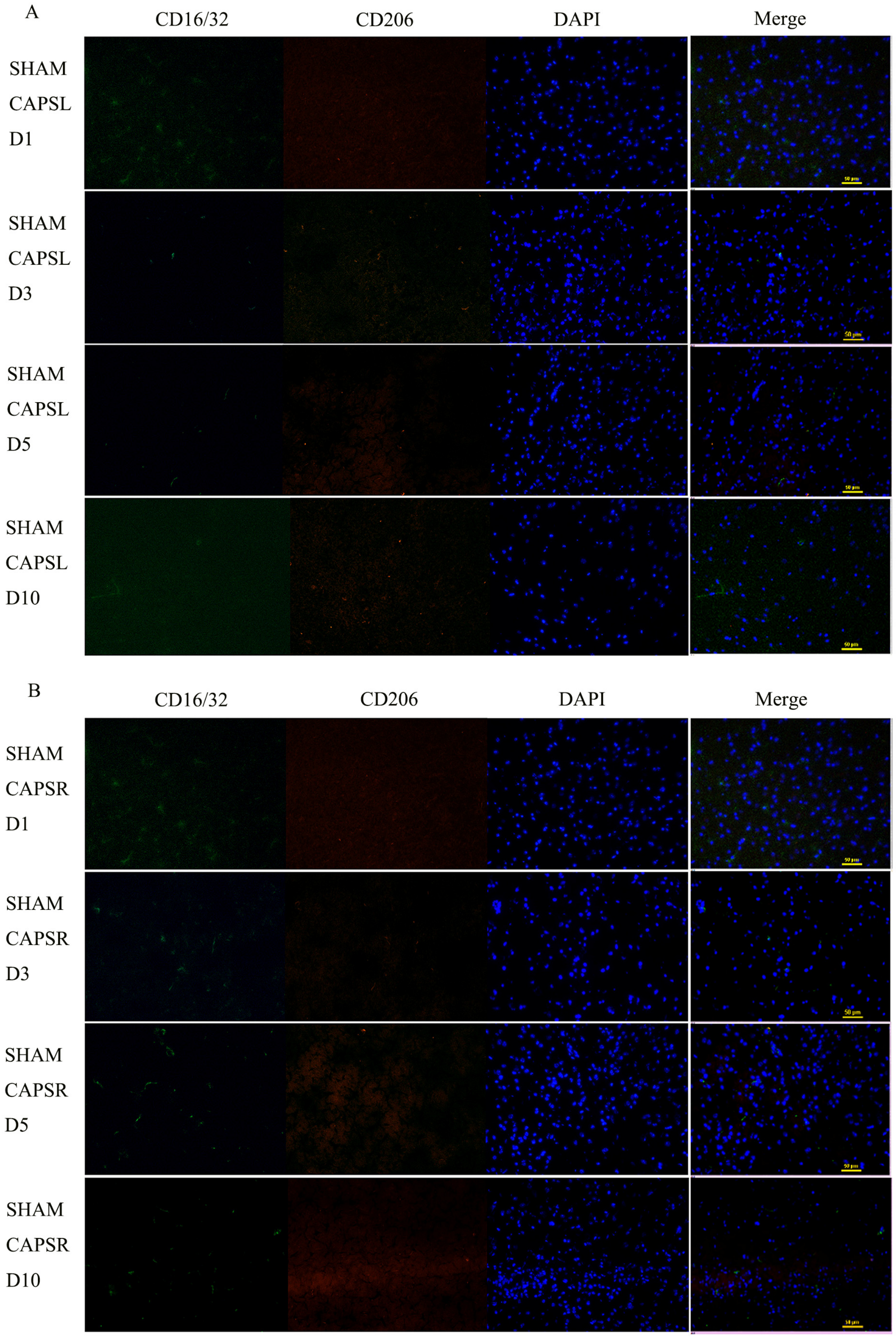


**Figure S3-** IF staining of CD16/32 (Green) and CD206 (Red), in D1, D3, D5 and D10

of SAHM group in CAPS left (A) and CAPS right (B). In shame group, we didn't see any change from M1 and M2 likes cell view.

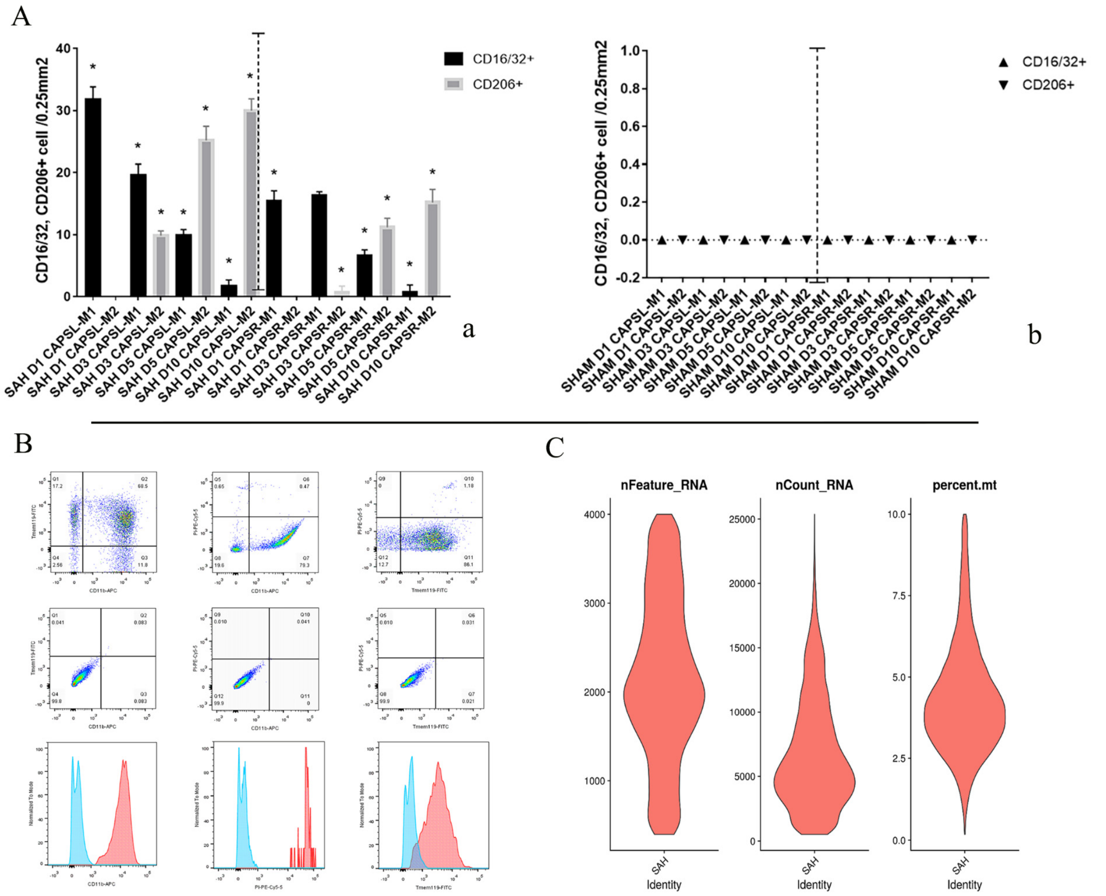


Final cell viability by hemocytometer 96.69%

Mitochondrial RNA correlation

**Figure S4-** A-B: Quantitative analysis of CD16/32 and CD206 positive cells in SAH (**A**) and sham group (**B**), the result shows CD206 positive cells gradually increase and CD16/32 positive microglia gradually decrease after SAH while there has no change in SHAM group. n=6-8, *p<0.05. C: Flow cytometry results of isolated cells shows that magnetic-activated cell sorting (MACS) of CD11b+ microglia with high purity (81.5%) and viability (97%); D: Mitochondrial RNA correlation results.

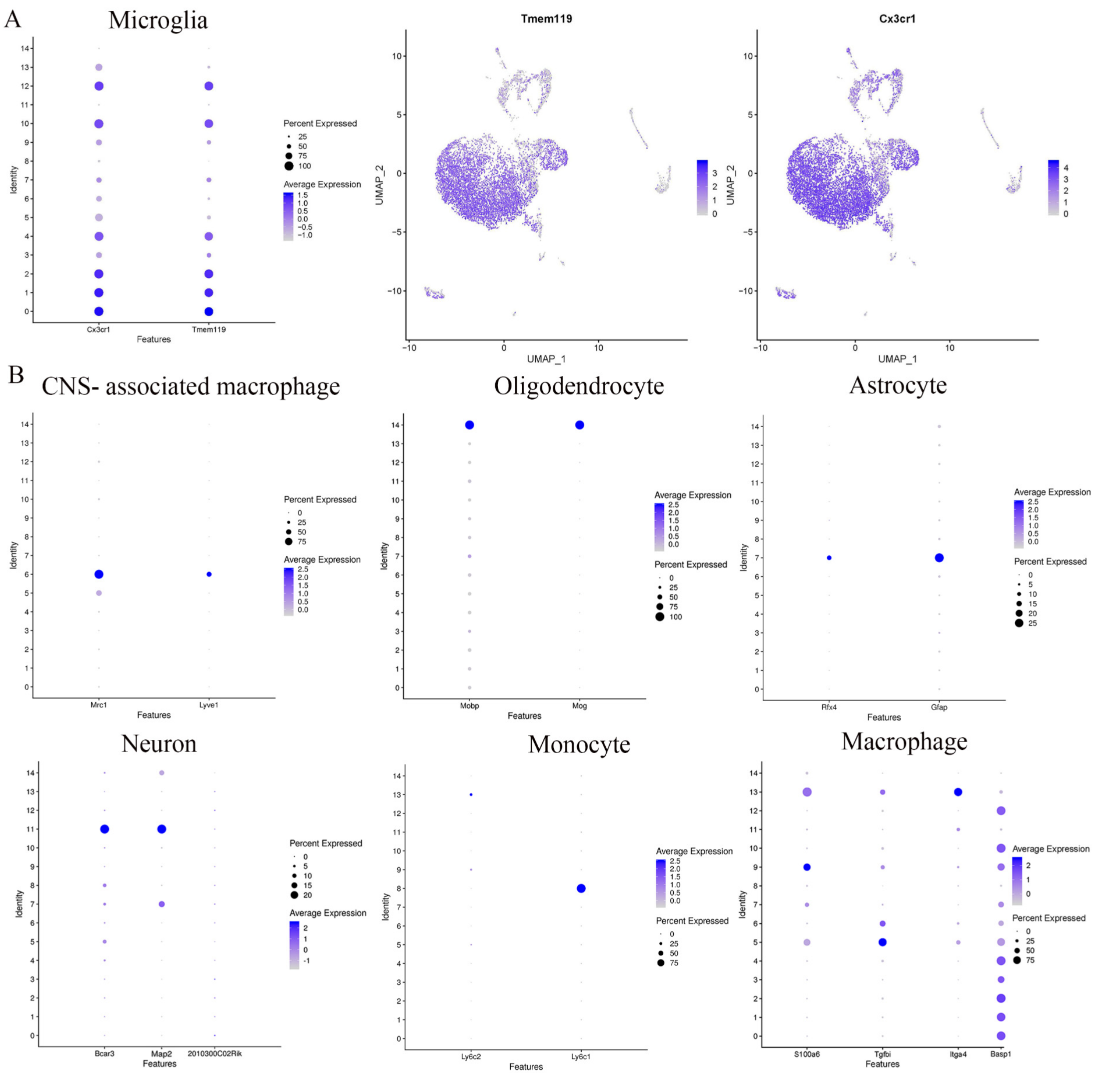


**Figure S5- A**: Microglial cell markers distribution (*Tmem119, Cx3cr1*); **B**: Based on cell type-specific marker gene expression patterns, we classified the single-cell transcriptomes into microglia (*n* = 5,824), central nervous system (CNS)-associated macrophages (*n* = 440), macrophages (*n* = 1,866), astrocytes (*n* = 301), monocytes (*n* = 249), neurons (*n* = 196) and oligodendrocytes (*n* = 40). Cell markers distribution of CNS- associated macrophage (*Mrc1, Lyve1*), oligodendrocyte (*Mobp. Mog*), astrocyte (*Rfx4, Gfap*), neuron (*Bcar3, Map2, 2010300C02Rik*), monocyte (*Ly6c1, Ly6c2*) and macrophage (*S100a6, Tgfbi, Itga4, Basp1*).

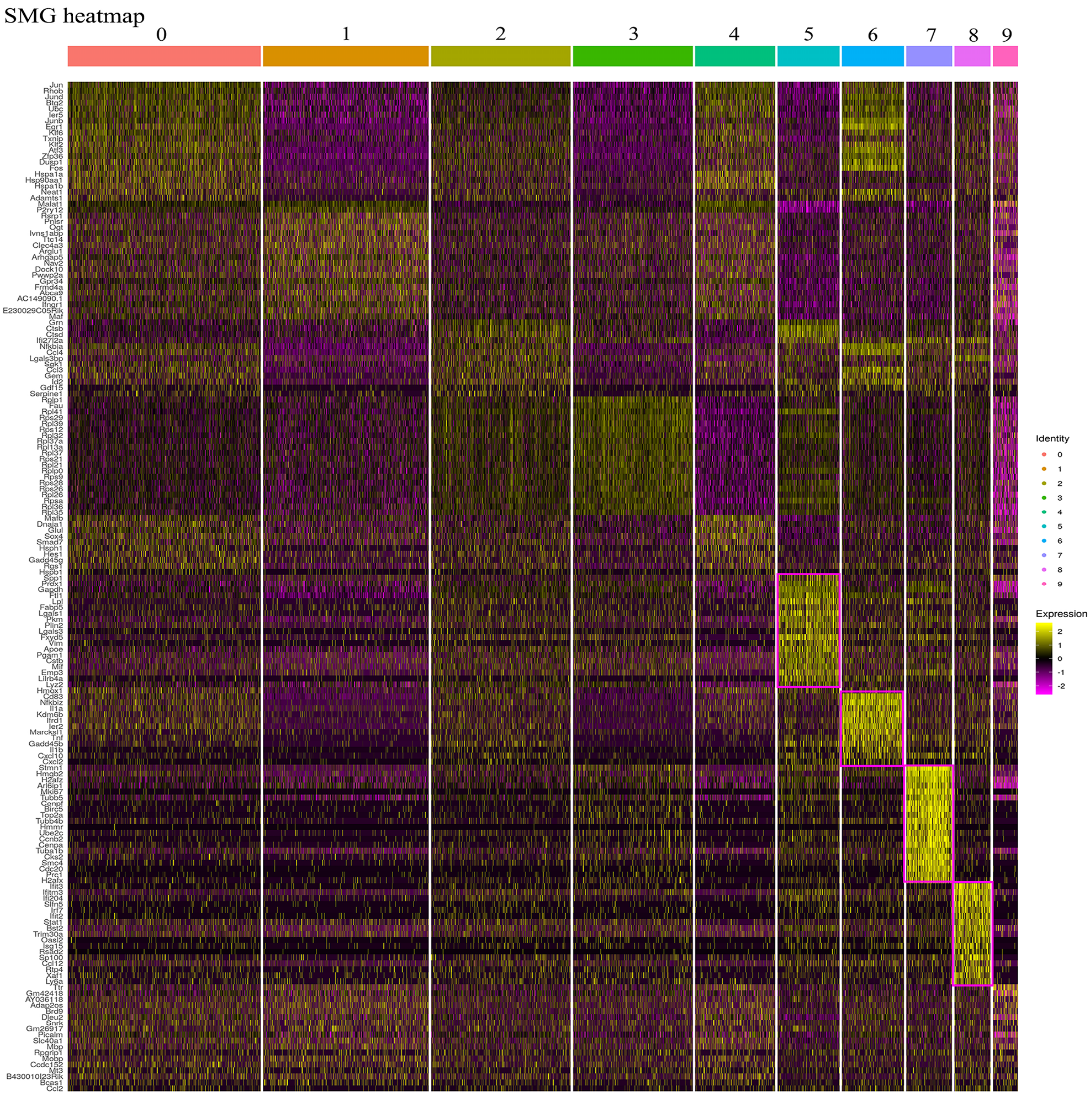


**Figure S6-** Gene expression of SAH microglia (SMG- heatmap). SMG cluster 0 (SMG-C0) was characterized by a high expression of immediate early genes encoding transcription factors (*Jun, Junb, Jund, Fos, Egr1, ler5, Klf6, Klf2 and Atf3 etc.*); SMG-C1 represented a microglial cluster that expresses high levels of homeostatic genes, including *P2ry12, P2ry13, Siglech, Gpr34, Selplg* and *Pmepa1*; SMG-C2 was characterized by high expression levels of *Ctsb*, *Ctsd*, *Ccl3*, *Ccl4, Lgals3bp*, *Cst7, Grn* and *Nfkbia etc;* In the SMG-C3 subpopulation, high expressions of ribosomal were found, including *Rplp1*, *Rplp0*, *Rpl41*, *Rps27a* and *Rps23 etc; S*MG-C4 has high transcript counts for genes encoding important transcription factors related to microglia reactive changes (e.g., *Mafb, Btg2* and *Txnip*). They also have high expression of microglial homeostasis genes (*P2ry12, Selplg* and *P2ry13*), HSP70 family and *Malat1*.

SMG-C5 was characterized by the expression of marker genes, including *Spp1, Lpl, Apoe, Ctsb, Lgals1, Lgals3, Fabp5, Mif, Lilrb4a, Lyz2, CD63, Cst7* and *Vim;* SMG-C6 microglia expressed a variety of cytokines (included *Il1a* (encoding interleukin 1 alpha), *Il1b* (encoding interleukin 1 beta), *Tnf* (encoding tumor necrosis factor α), *Ccl4* and *Ccl3*), chemokines (including *Cxcl10* (C-X-C Motif Chemokine Ligand 10), *Cxcl2* (C-X-C Motif Chemokine Ligand 2)), and other immune-signalling regulatory genes (including *CD83, CD74* (MHC class II), *CD14*, *Nfkbia* and *Nfkbiz* (NF-κB inhibitor Zeta)); SMG-C7 have high expression of proliferative and cell cycle genes (e.g., *Birc5* (baculoviral IAP repeat-containing 5), *Ccnb2*, *Cenpa, Cenpf, Mcm5, Ube2c, H2afz*, *H2afx, Cdks and Mki67*); SMG-C8 marker genes were characterized by several interferon responsive genes (including *Ifit3, Ifitm3, Ifi204, Slfn5, Irf7, Ifit2, Ifi27l2a* and *Cxcl10*); SMG-C9 abundantly expressed *Ttr, Mbp, Mobp, Myh9, Slc40a1* and *Dennd1c*.

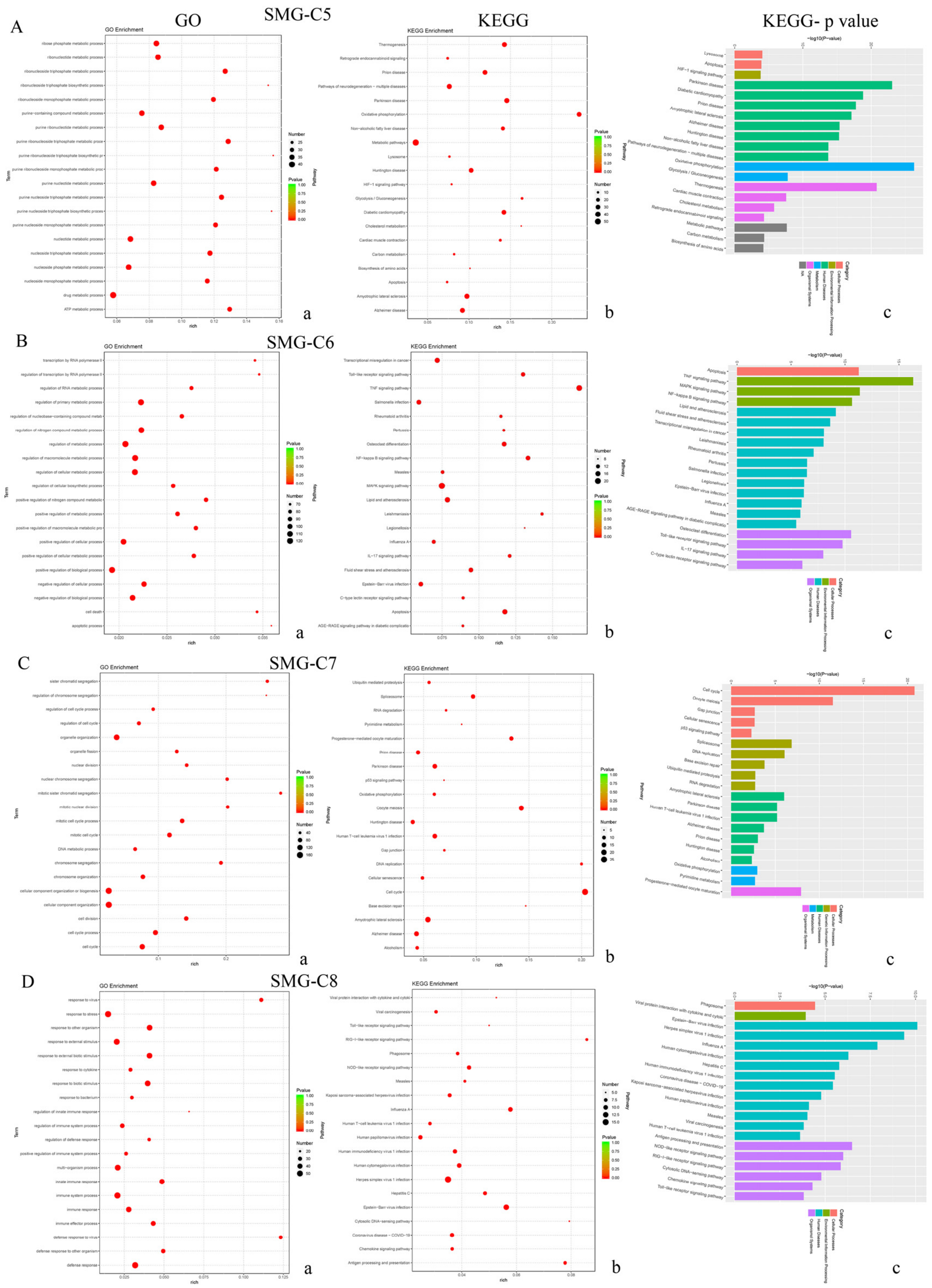


**Figure S7-** Enrichment analysis of SAH microglia (SMG). **A**: SMG-C5, the upregulated genes in SAM are related to oxidative phosphorylation, lysosome, apoptosis, glycolysis/gluconeogenesis etc. **B**: SMG-C6, SMG-C6 were enriched in genes related to interleukin, tumor necrosis factor (TNF), toll-like receptor 4 (TLR4)

and nuclear factor-kappa B (NF-κB) signaling pathways etc. **C**: SMG-C7, Pathway enrichment results indicated that SMG-C7 was enriched with genes associated with mitotic nuclear division, cell division and cell cycle etc. **D**: SMG-C8, SMG-C8 was suggested to be highly responsive to cytokines and other biological stimuli, potentially mediated by the MHC class I protein complex, chemokine activity regulation and CCR chemokine receptor binding a: GO, b-c: KEGG.

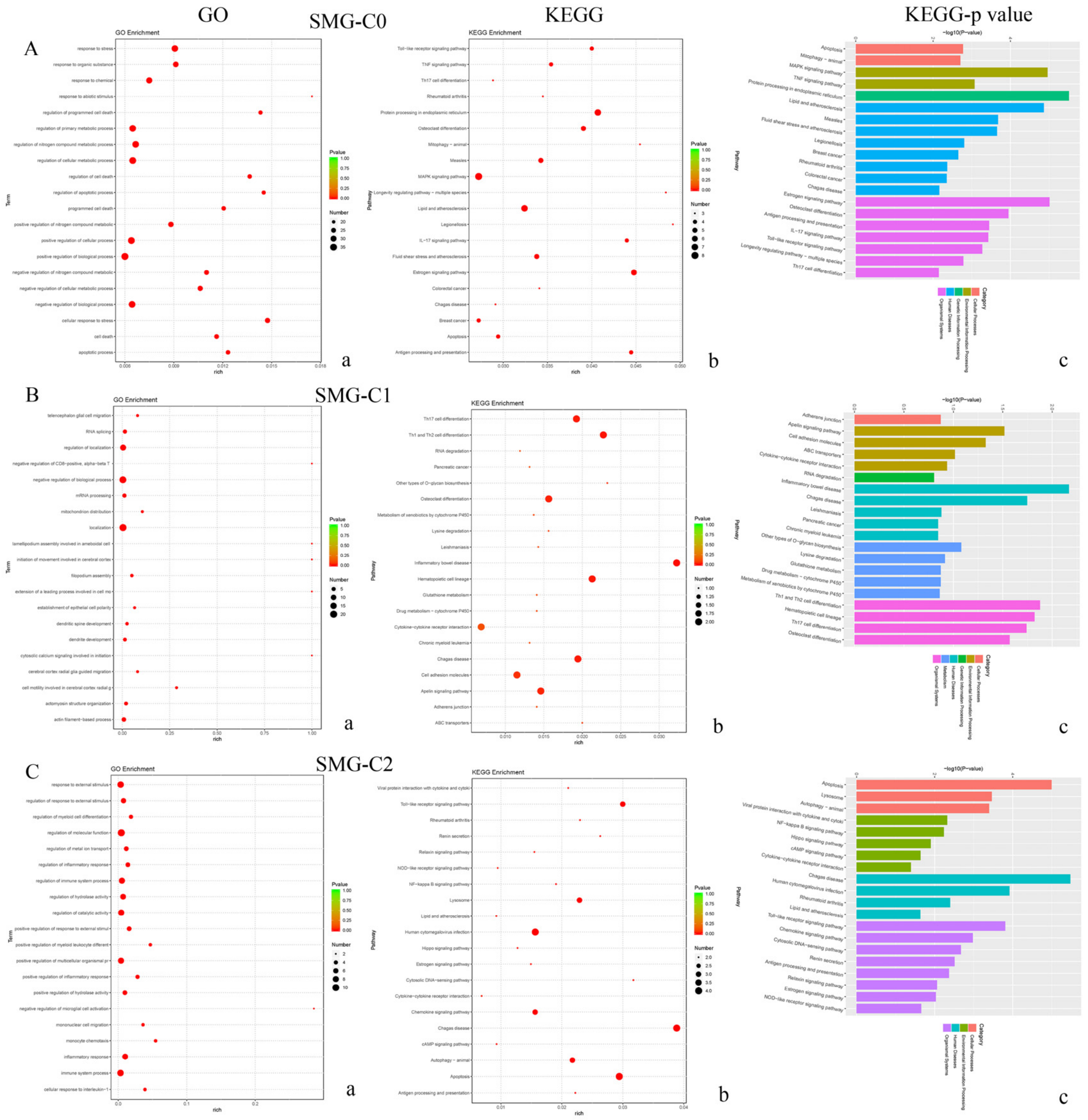


**Figure S8-** Enrichment analysis for SMG microglia. **A**: SMG-C0, **B**: SMG-C1 and **C**: SMG-C2; a: GO and b-c: KEGG. SMG-C0 was enriched with the negative regulation of the cellular metabolic process, regulation of the apoptotic process, cellular response to stress and response to organic substances; SMG-C1 with homeostatic functions; SMG-C2 transcriptomes were enriched in genes involved in regulating metal ion

transport, monocyte chemotaxis, regulation of inflammatory response and regulation of hydrolase activity.

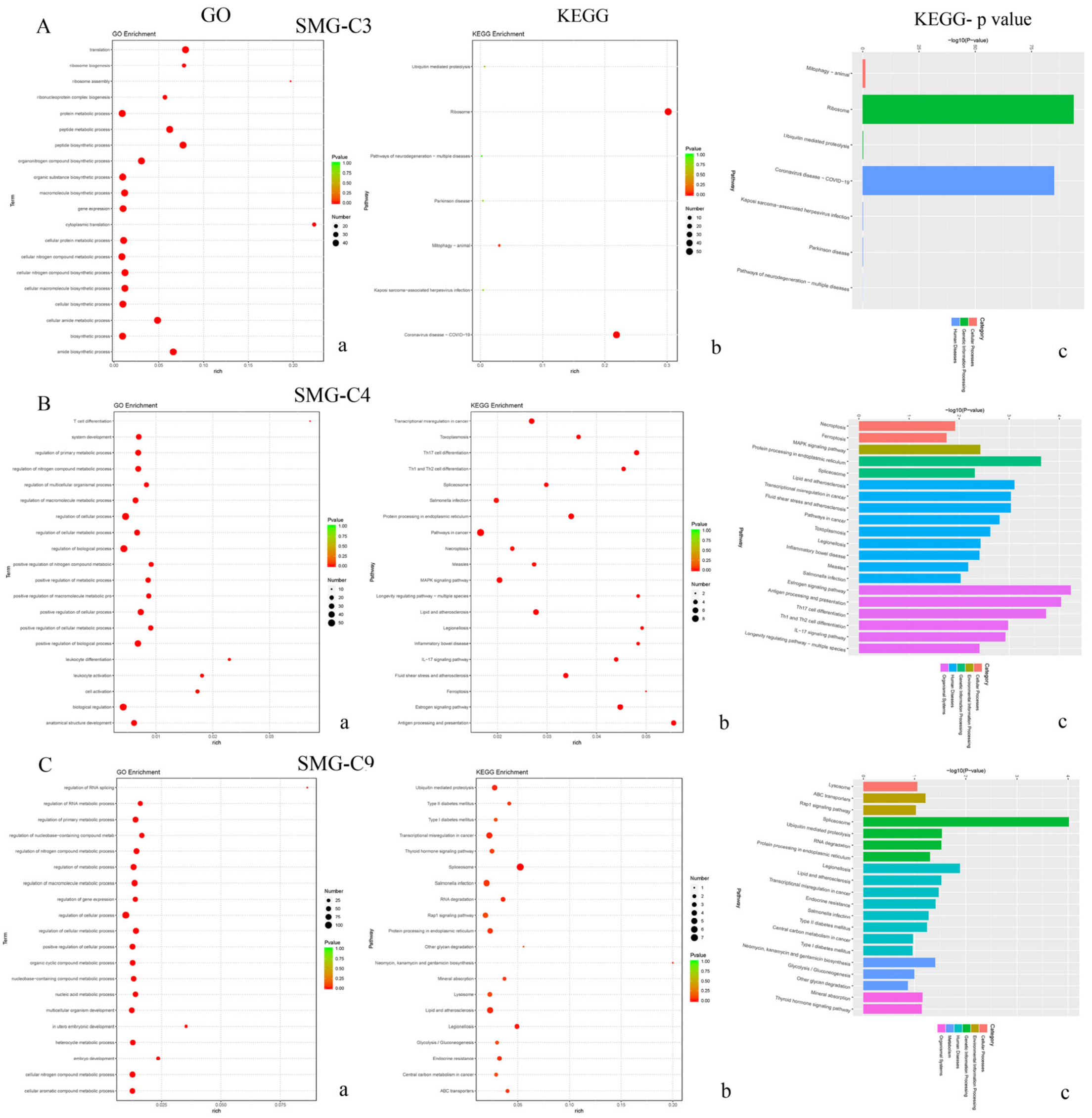


**Figure S9-** Enrichment analysis for SMG microglia. **A**: SMG-C3, **B**: SMG-C4 and **C**: SMG-C9. a: GO and b-c: KEGG. SMG-C3 were connected with ribosome functions; the SMG-C4 highly expressed genes were found related to leukocyte activation, positive regulation of cellular metabolic process and positive regulation of nitrogen compound metabolic process; SMG-C9 was enriched with genes related to the regulation of the metabolism, RNA metabolic processes, lysosomes and ABC transporters.

Top 10 markers in SMG-C5

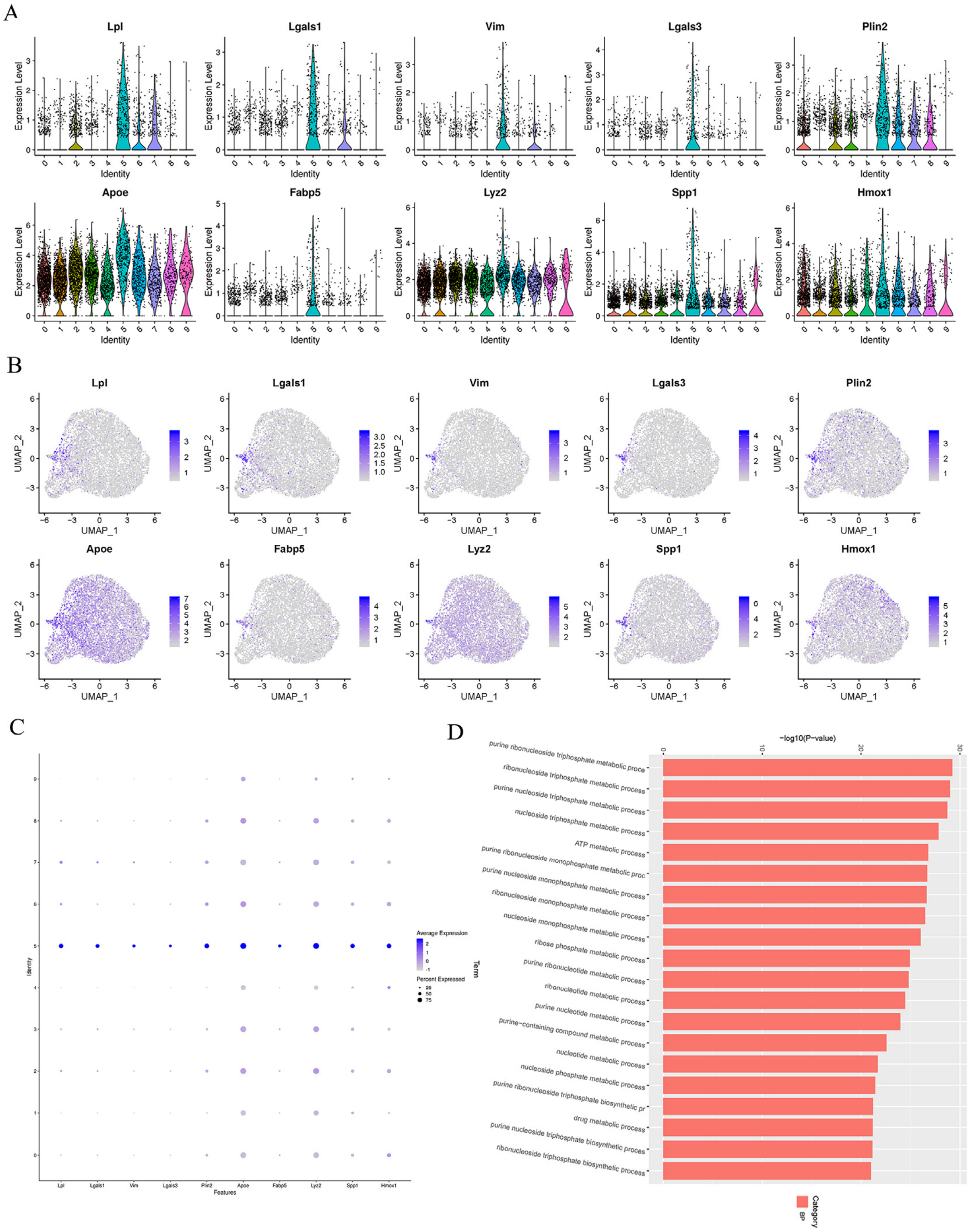


**Figure S10: A**: Violin atlas of top 10 genes in SAH microglia cluster 5 (SMG-C5)-SAM; **B**: Top 10 genes distribution in SMG-C5; **C**: Top 10 genes expression dot plot in SMG-C5; **D**: Enrichment of SMG-C5.

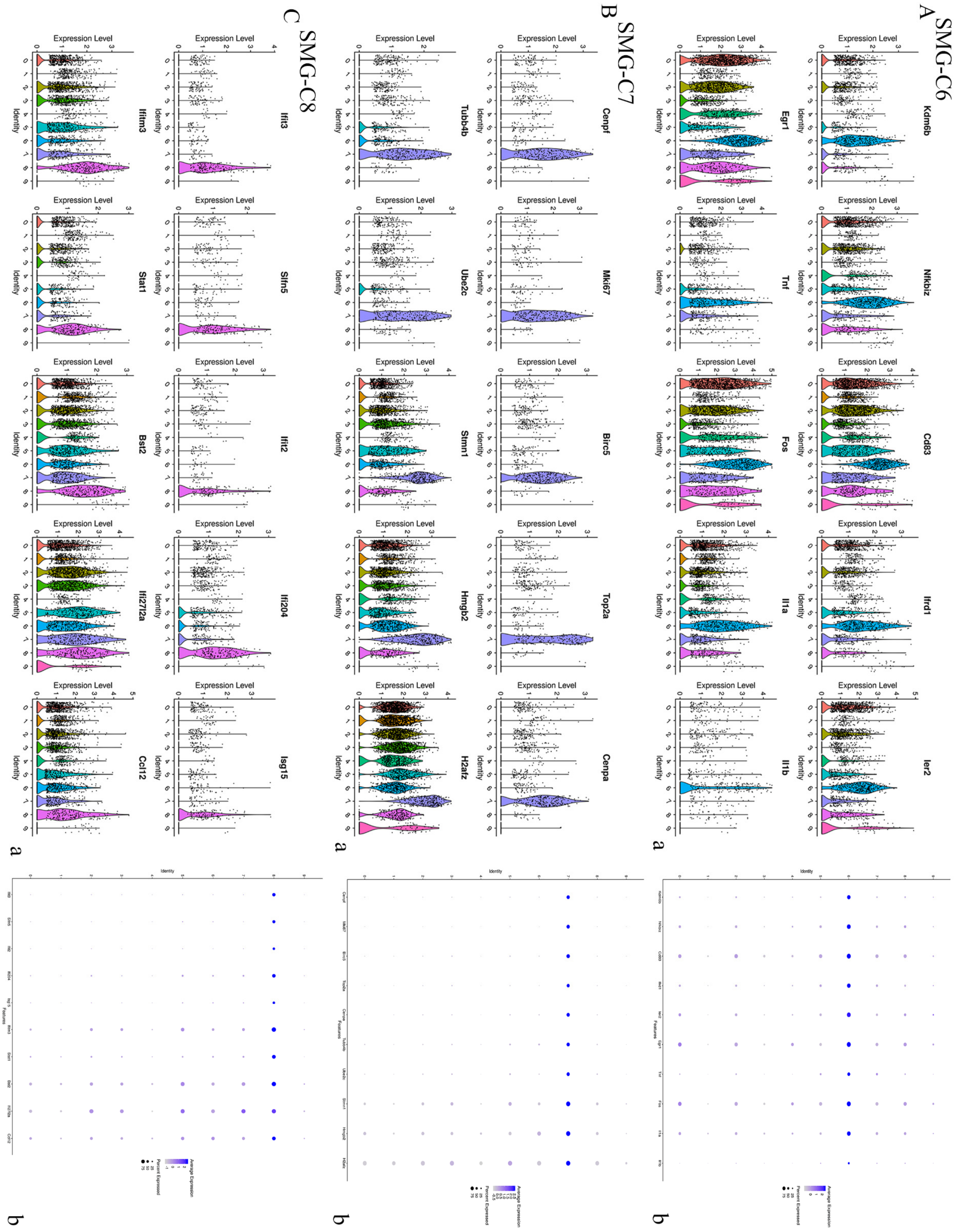


**Figure S11-** Top 10 expressed genes in SMG microglia- Atlas of violins and dot plot.

**A**: SMG-C6, **B**: SMG-C7 and **C**: SMG-C8.

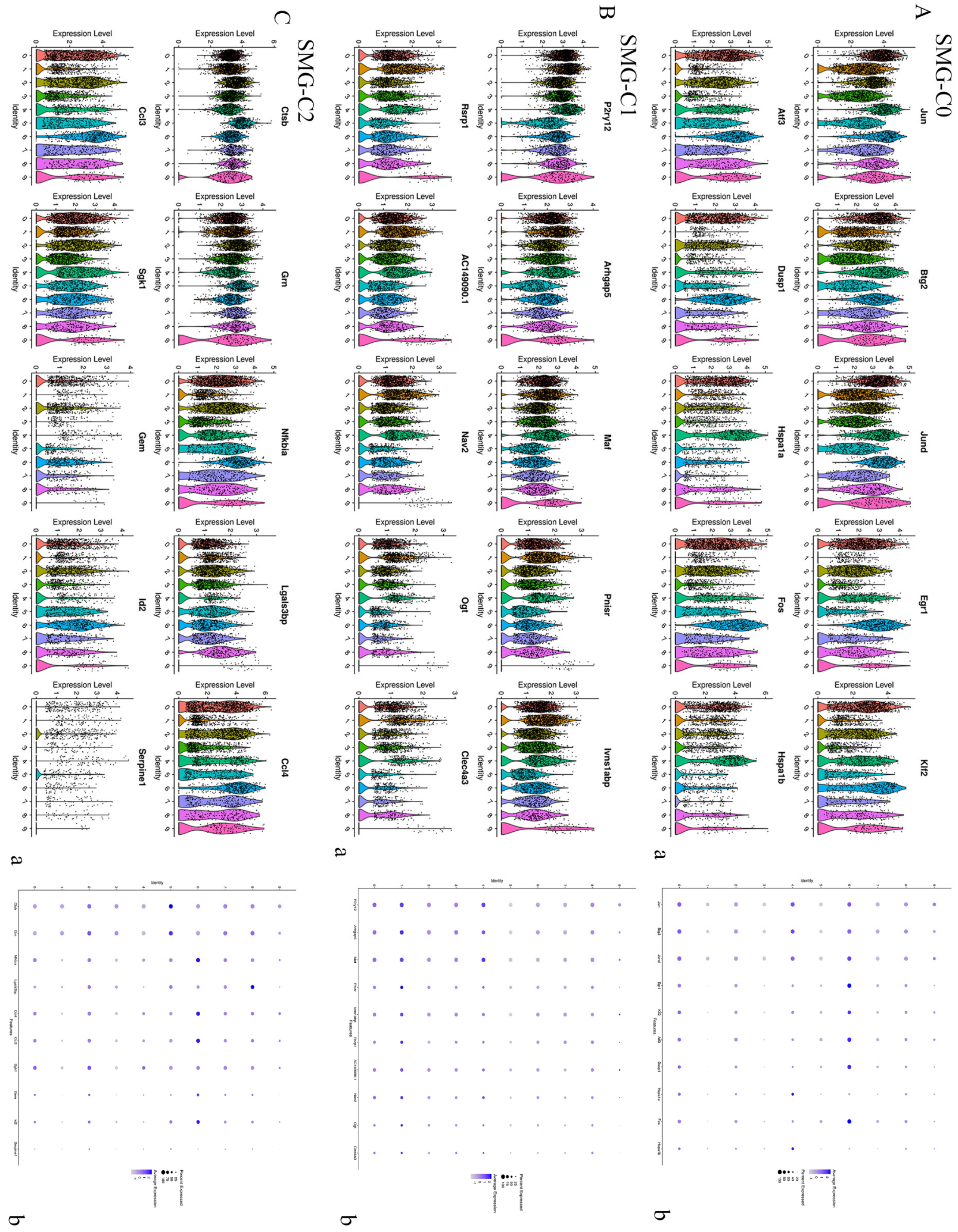


**Figure S12-** Top 10 expressed genes in SMG microglia- Atlas of violins and dot plot.

**A**: SMG-C0, **B**: SMG-C1 and **C**: SMG-C2.

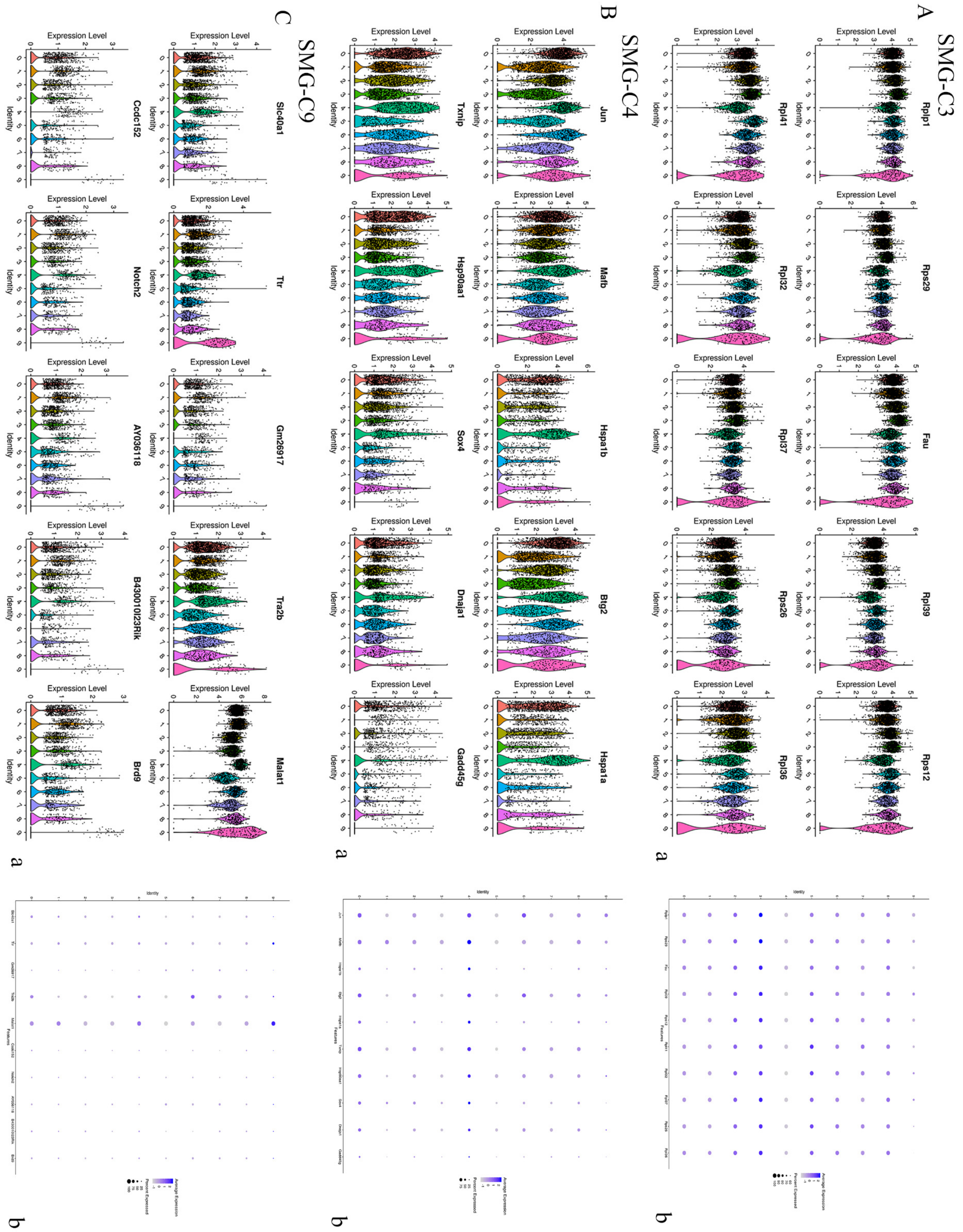


**Figure S13-** Top 10 expressed genes in SMG microglia- Atlas of violins and dot plot.

**A**: SMG-C3, **B**: SMG-C4 and **C**: SMG-C9.

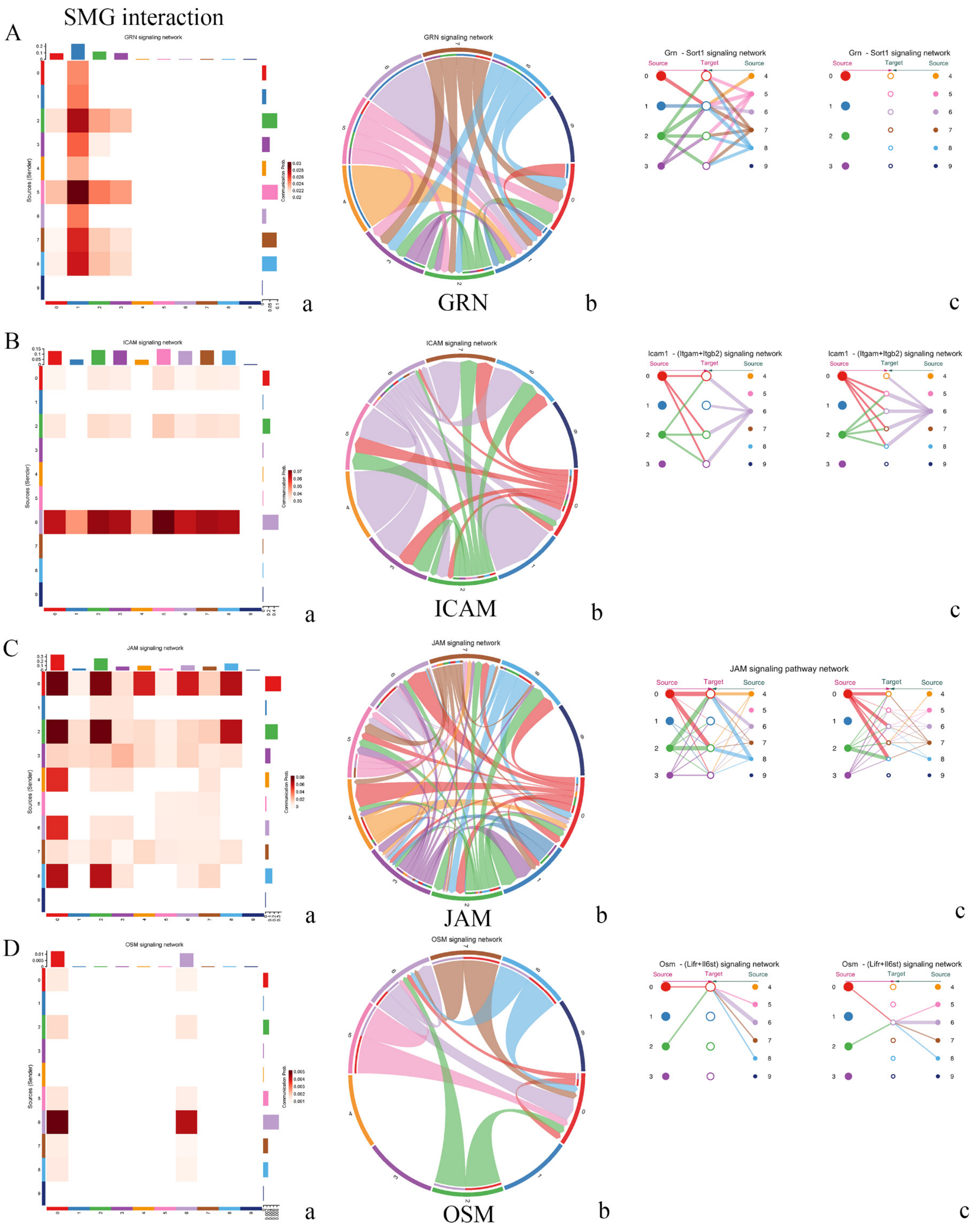


**Figure S14-** SMG microglia subsets interaction (signaling pathway). **A**: GRN, **B**: ICAM, **C**: JAM and **D**: OSM signaling pathway. a: heatmap, b: chord map and c: hierarchy connection.

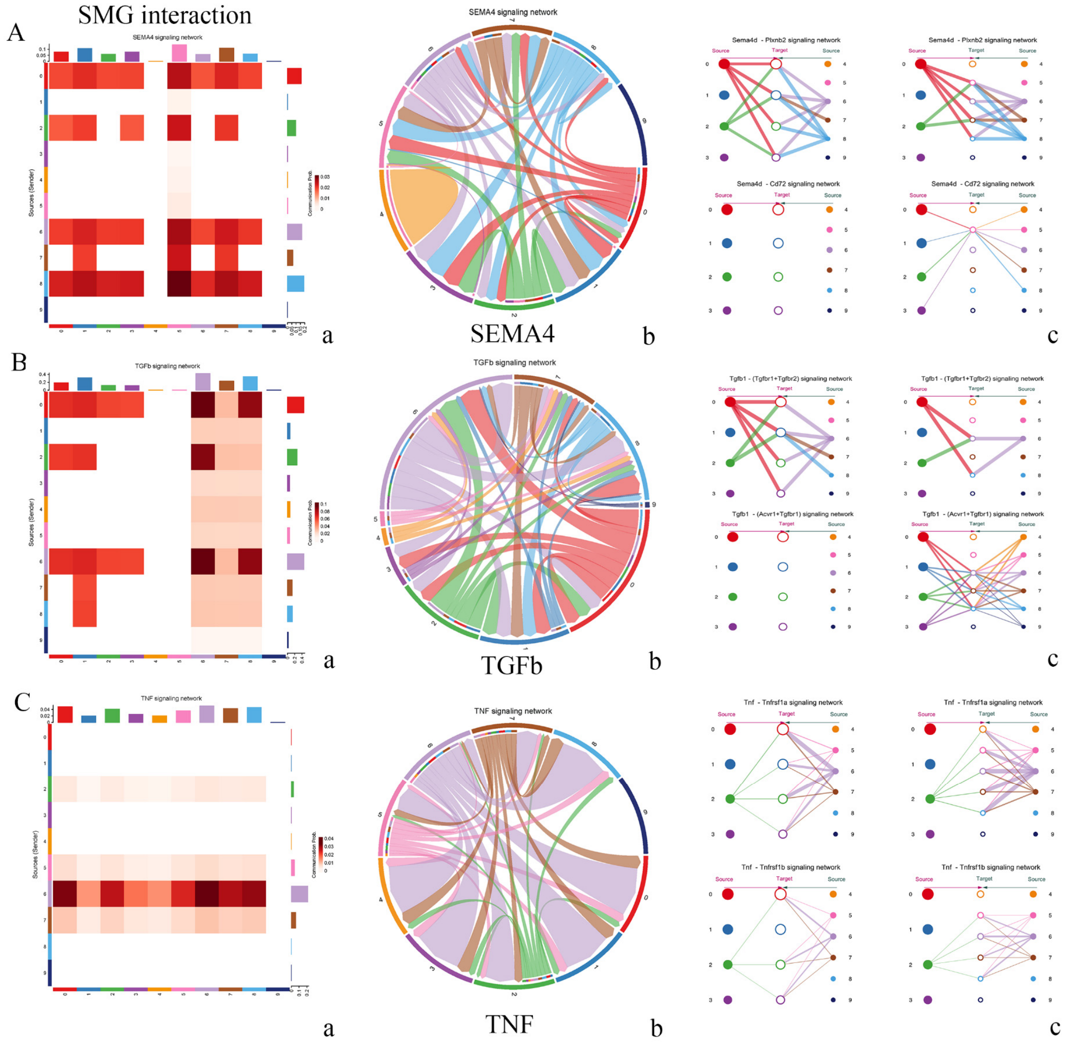


**Figure S15-** SMG microglia subsets interaction (signaling pathway). **A**: SEMA4, **B**: TGFb and **C**: TNF signaling pathway. a: heatmap, b; chord map and c: hierarchy connection.

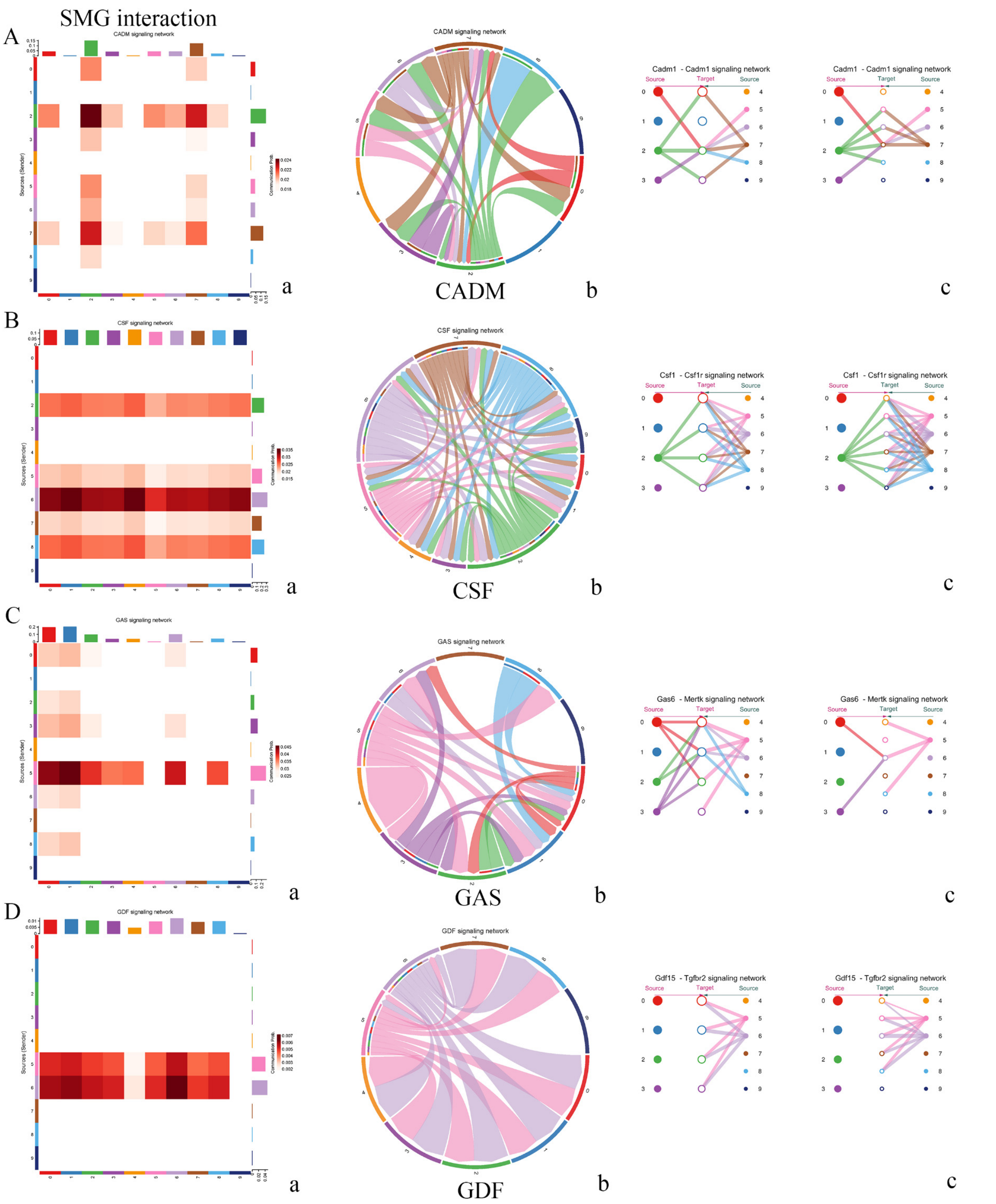


**Figure S16-** SMG microglia subsets interaction (signaling pathway) **A**: CADM, **B**: CSF, **C**: GAS and **D**: GDF signaling pathway. a: heatmap, b; chord map and c: hierarchy connection.

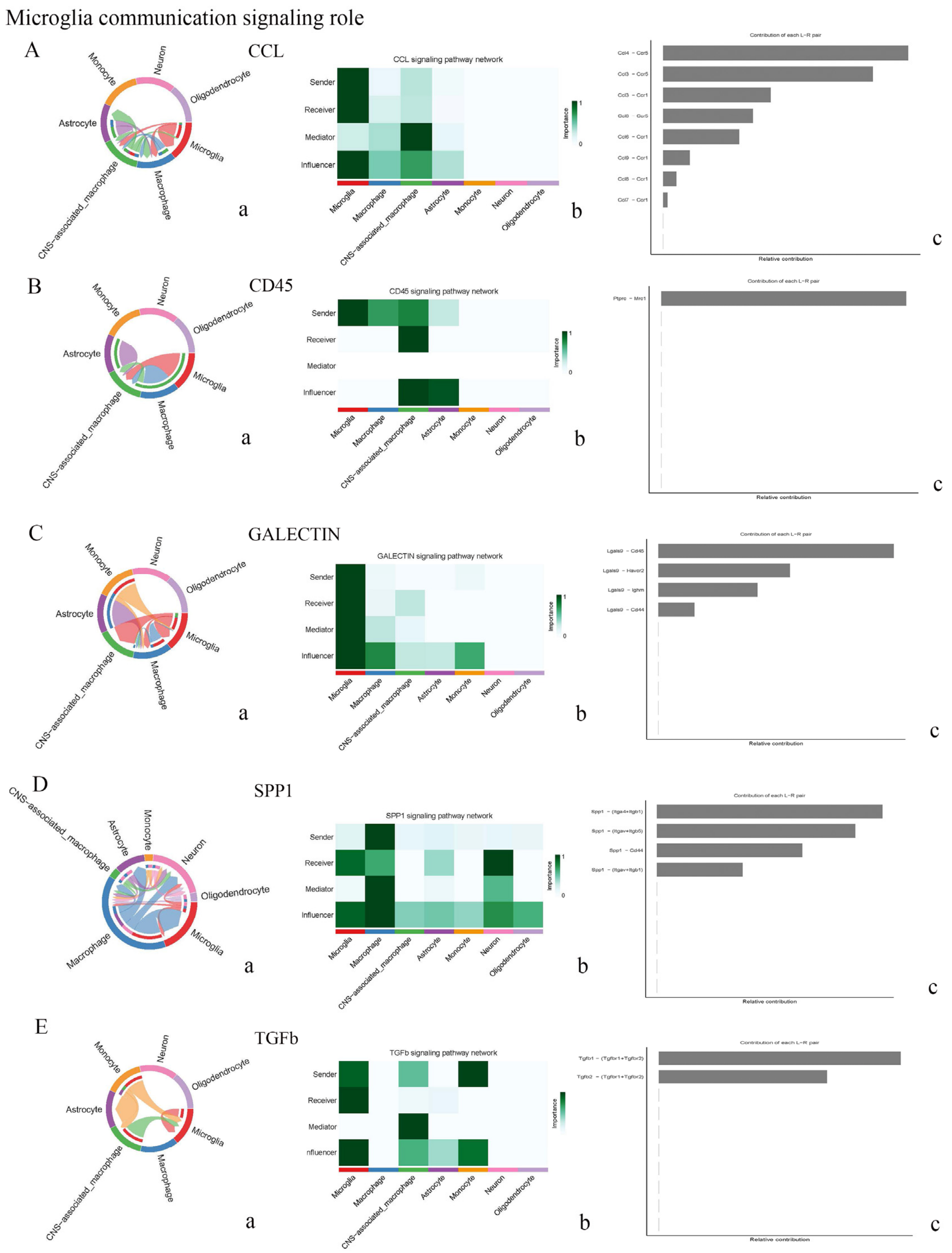


**Figure S17-** Signaling pathways expressions- microglia interacts with seven types of cells. **A**: Microglia is the mainly sender and receiver of CC-motif chemokine ligand (CCL) signaling pathway, and CNS-associated macrophage is important mediator, other cells not join; **B**: Microglia is the mainly sender and CNS-associated macrophage is the mainly receiver of CD45; **C**: GALECTIN signaling pathway mainly depended on microglia; **D**: Microglia and Neuron cell are the mainly receiver of SPP1, while

macrophage is the only sender; and **E**: Microglia is the only receiver and monocyte is the sender of TGFb, while CNS-associated macrophage is the mediator; a: chord map, b: contributing role and c: signaling role hierarchy.

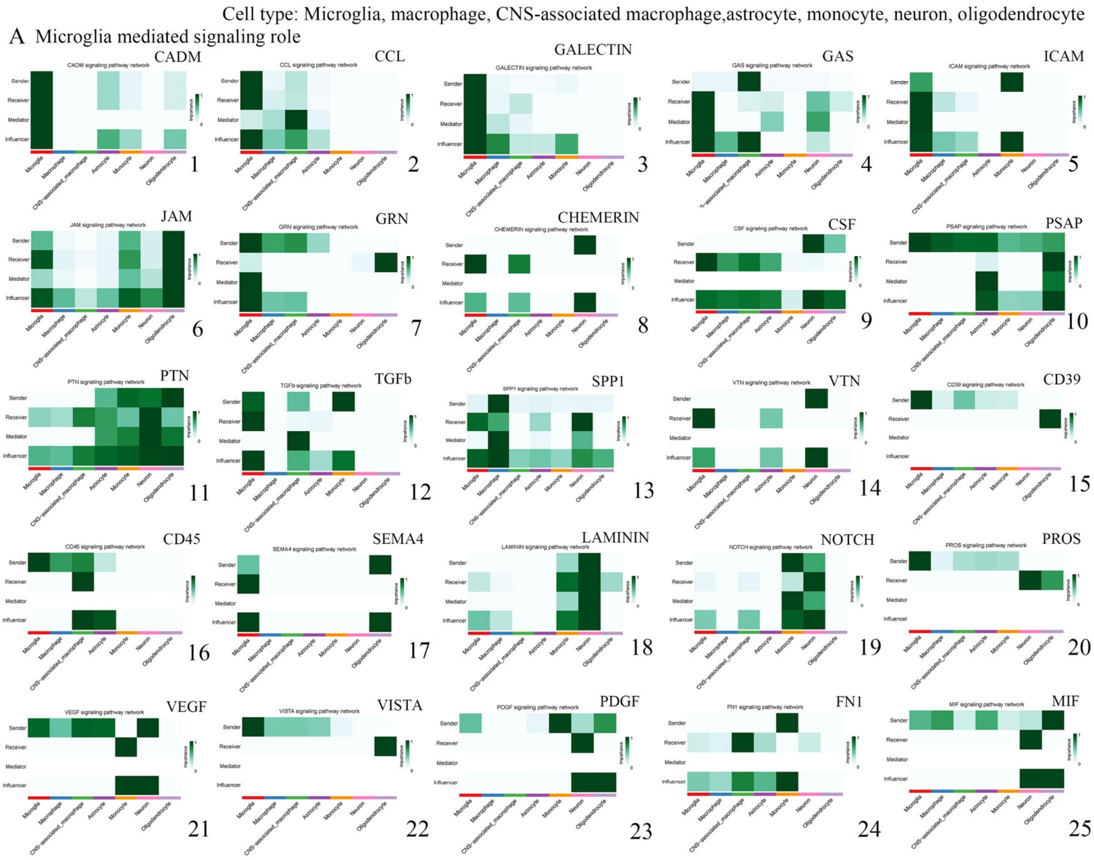


**Figure S18-** Microglia interacts with Microglia, macrophage, CNS-associated macrophage, astrocyte, monocyte, neuron and oligodendrocyte, Major contributing signaling role. **A**: microglia participated signaling pathway. Microglia have been found to participate in most post-SAH inflammatory pathways (25 signaling pathways in total), among which 13 signaling pathways involve interactions with neurons **(**including GAS, JAM, CHEMERIN, CSF, PSAP, PTN and SPP1-mediated pathways)

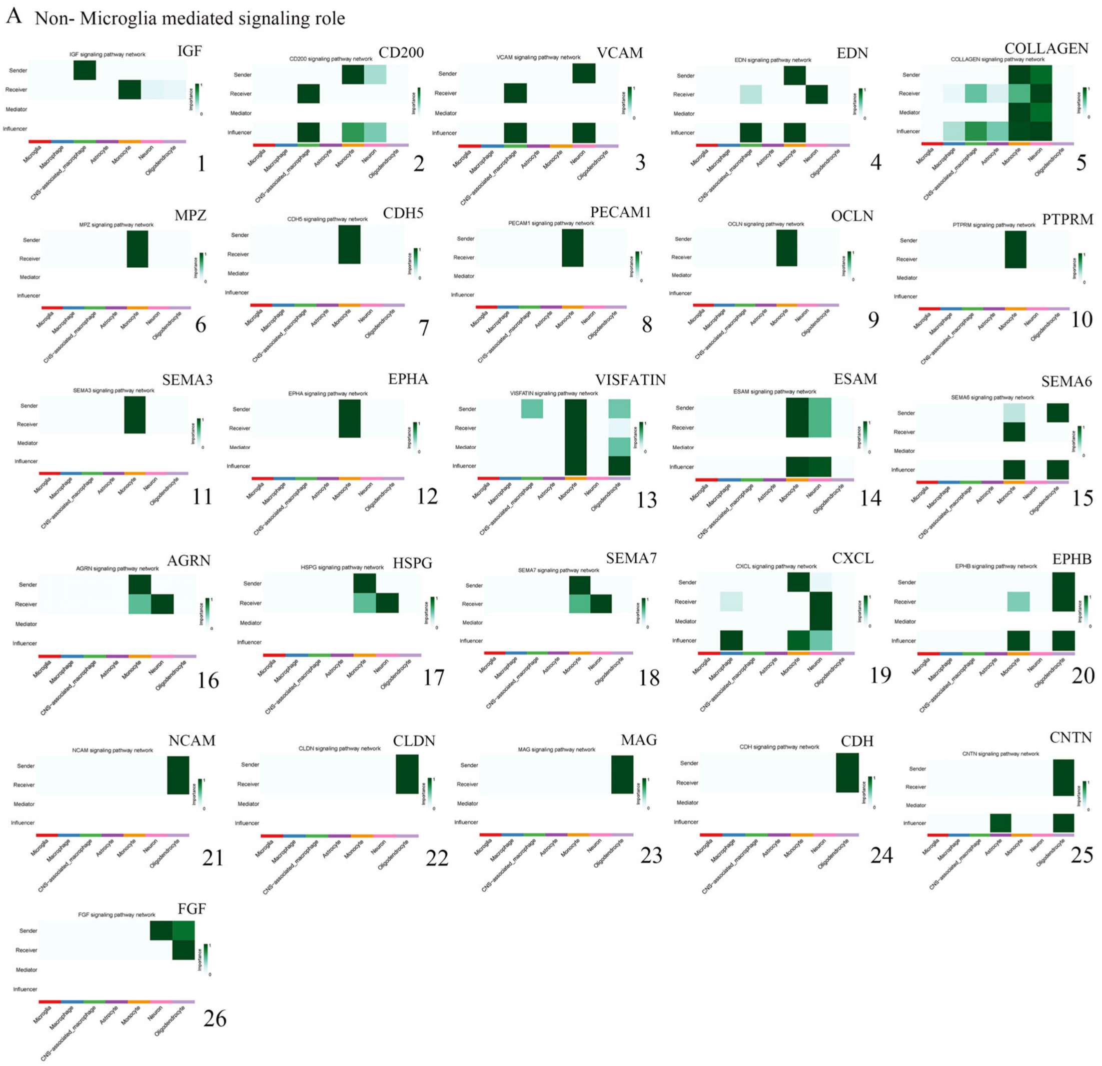


**Figure S19-** Microglia interacts with Microglia, macrophage, CNS-associated macrophage, astrocyte, monocyte, neuron and oligodendrocyte- Major contributing signaling role. **A**: None-microglia participated signaling pathway.

Microglia mediated signaling pathway expression

A

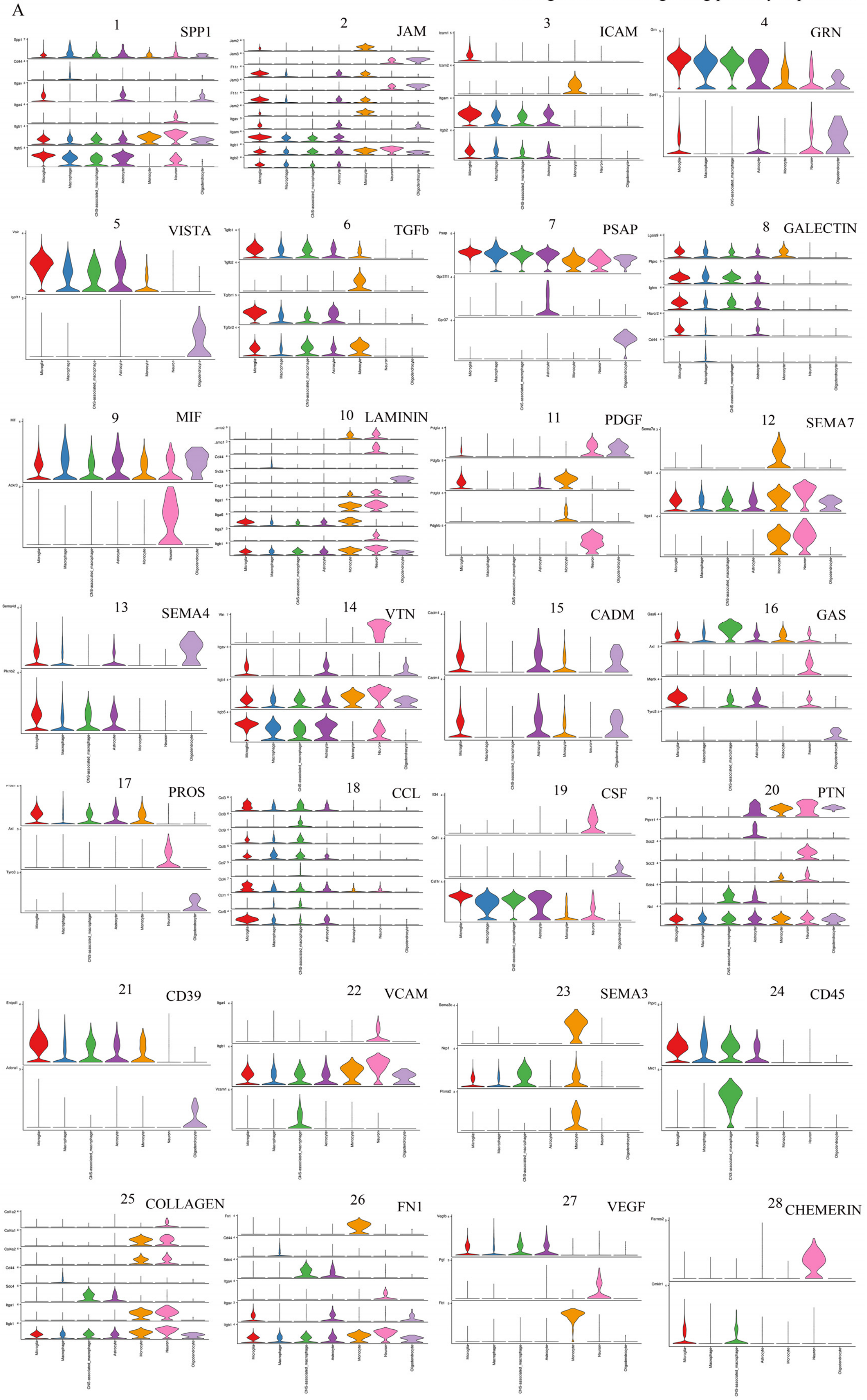

**Figure S20-** Microglia interacts with Microglia, macrophage, CNS-associated macrophage, astrocyte, monocyte, neuron and oligodendrocyte- Expression plot of signaling pathway. **A**: microglia participated signaling pathway.

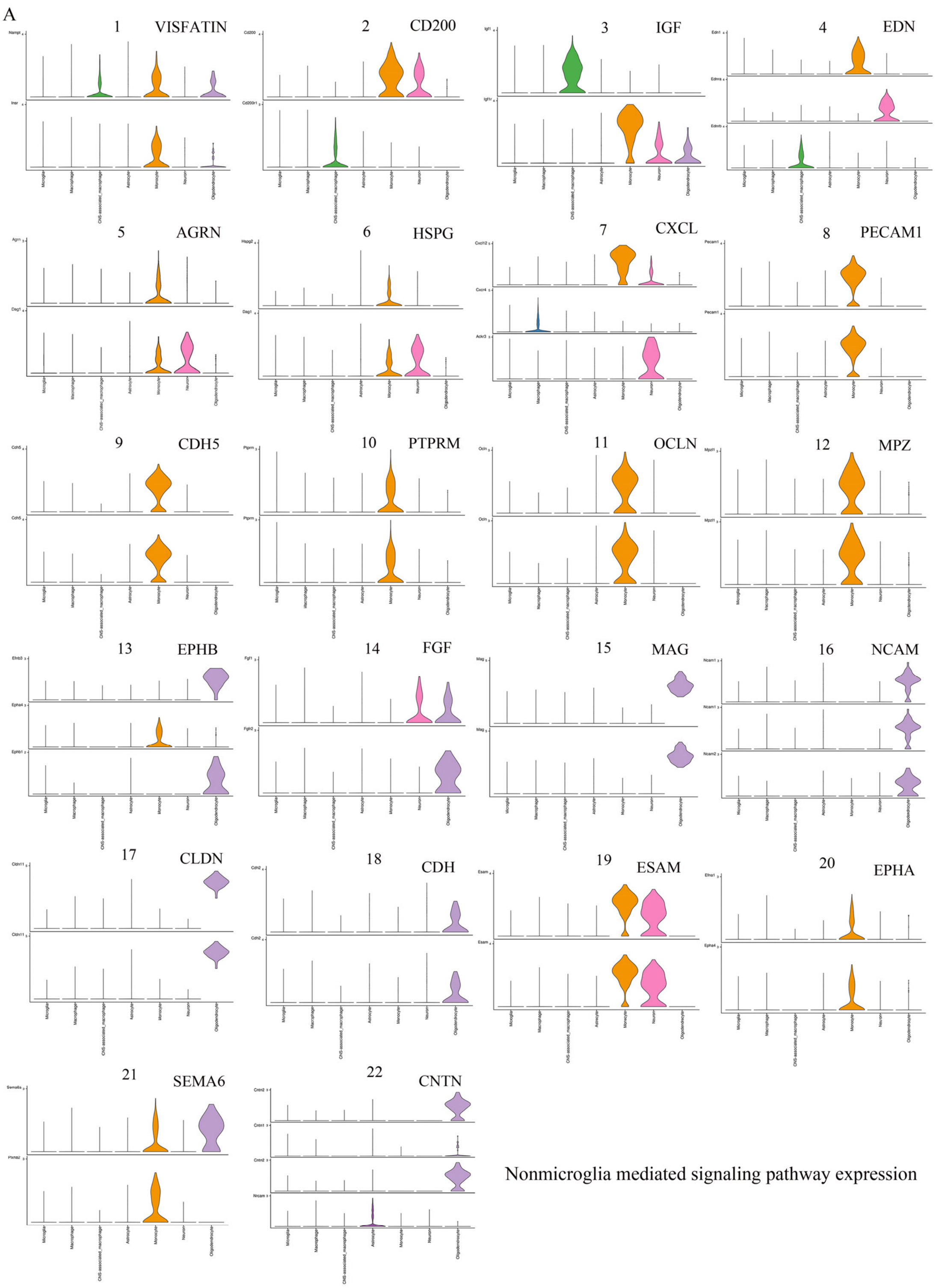


**Figure S21-** Microglia interaction with Microglia, macrophage, CNS-associated macrophage, astrocyte, monocyte, neuron and oligodendrocyte- Expression plot of

signaling pathway. **A**: Non-microglia participated signaling pathway.

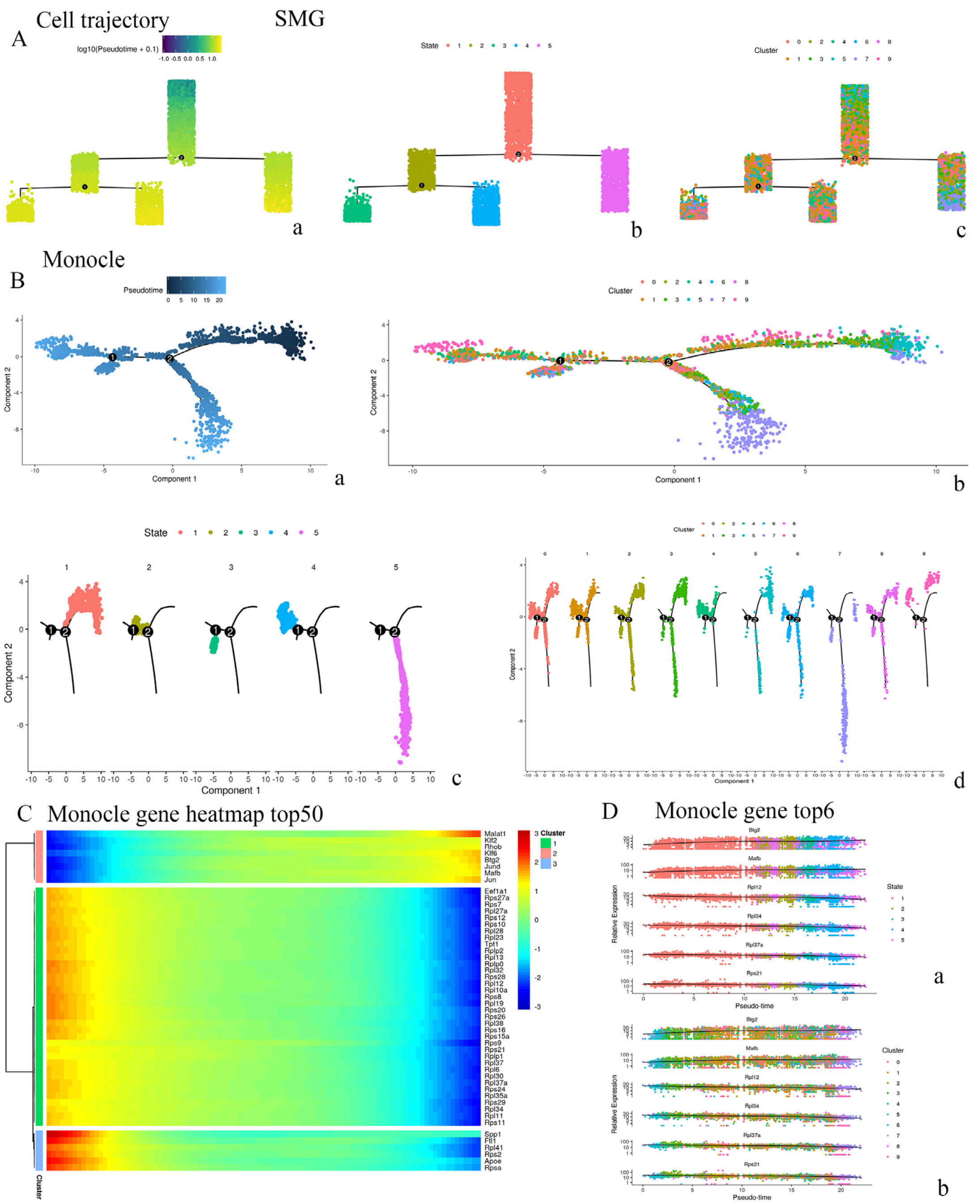


**Figure S22:** Trajectory analysis of SMG (10 clusters). **A**: Microglia trajectory found 5 states of post-SAH microglia; **B**: With monocle analysis, we noted that all subpopulations contain a variety of cell states. SMG-C4 and SMG-C9 were probably in an intermediate stage because they mainly contained states 1 and 4, but not state 5. The entire process of 5 states can be found in SMG-C2, SMG-C6 and SMG-C8. SMG-

C5, SMG-C6, SMG-C7 and SMG-C8 included the late states of microglia transformation Monocle branch (a: Pseudotime, b: monocle branch (cluster), c: monocle branch (state), d: monocle branch (cluster split)); **C**: monocle genes heatmap- From the heatmap of top 50 genes with pseudotime, the immediate early genes (*Jun, Jund, Klf6, Klf2* - enriched in SMG-C0), transcriptional regulatory genes (*Btg2, Rhob* and *Mafb* - enriched in SMG-C4) and *Malat1* were all upregulated with pseudotime; **D**: monocle genes state.

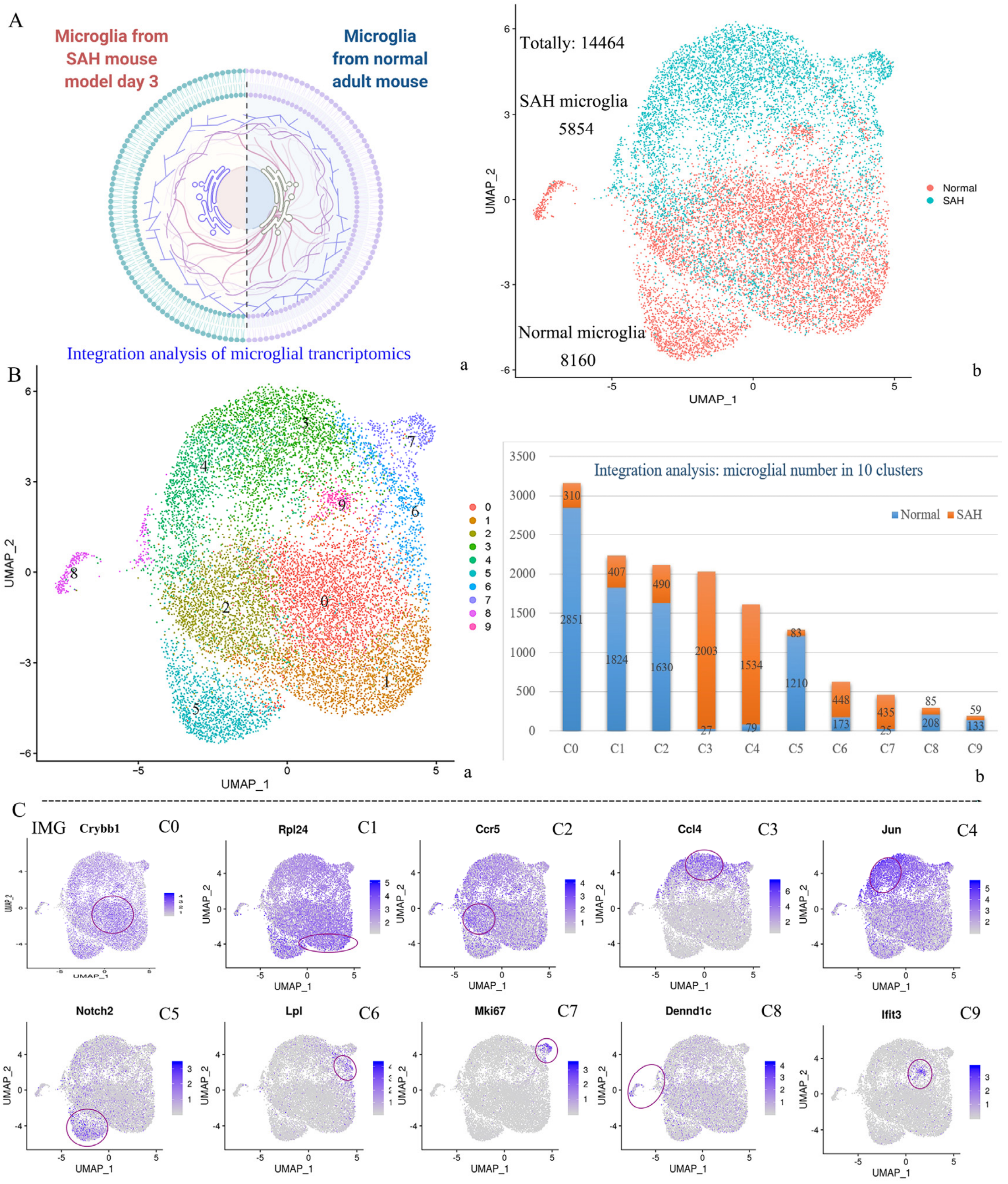

**Figure S23:** **A**: a-and b-Integrated analysis of SAH and normal samples with totally 14014 microglia cells (SAH (*n* = 5,854) and normal (*n* = 8,160)); **B**: a-UMAP of Integration analysis of SAH and normal microglia (IMG)-10 clusters, b-SAH and normal microglia numbers in each IMG cluster; **C**: Biomarkers of each clusters in IMG.

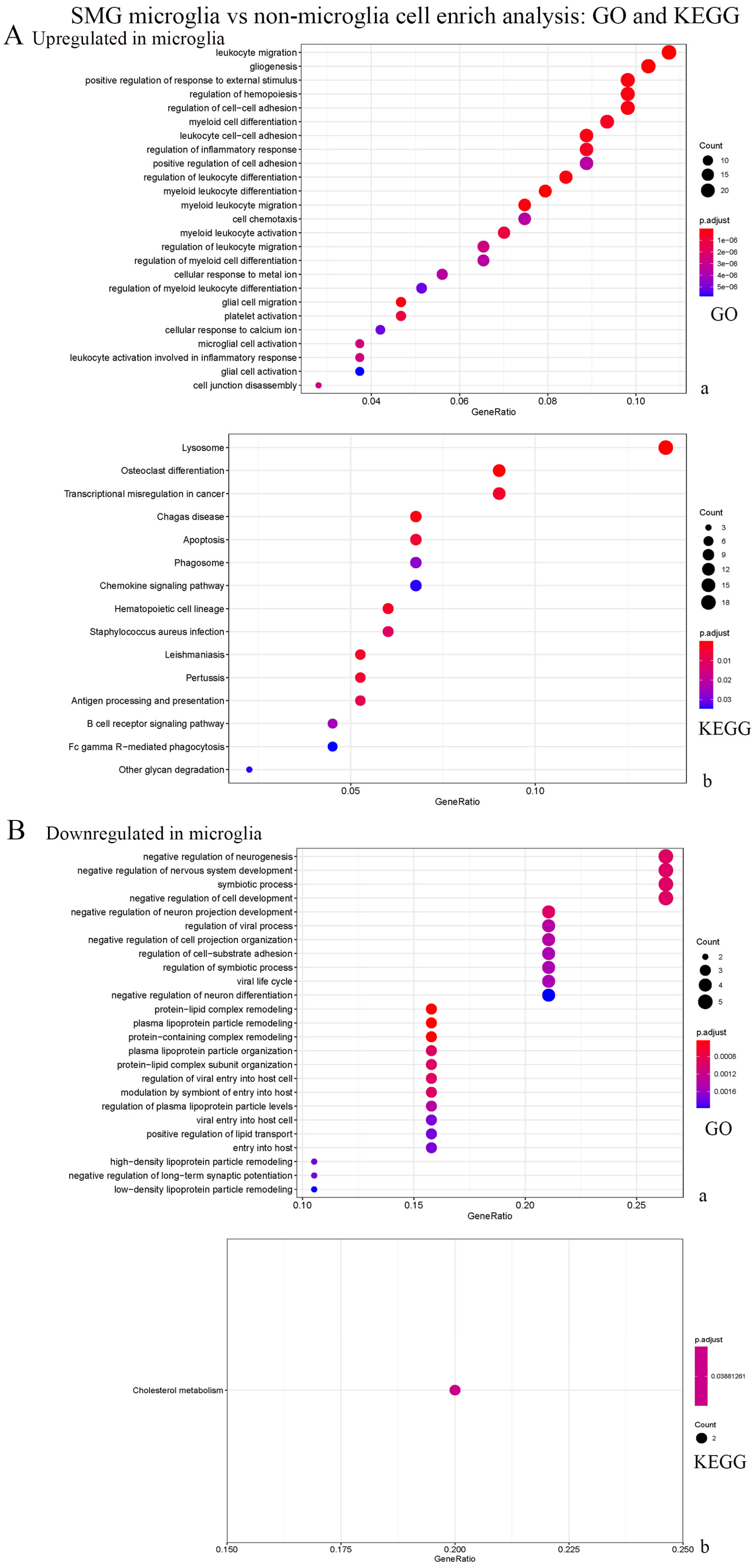


**Figure S24-** Enrichment of SMG Microglia vs non microglia cells. **A**: Upregulated

signaling pathway in SMG including leukocyte activation, migration and adhesion, glia cell migration and activation, gliogenesis, regulation of inflammatory response, lysosomal pathways; **B**: Downregulated signaling pathway in SMG.

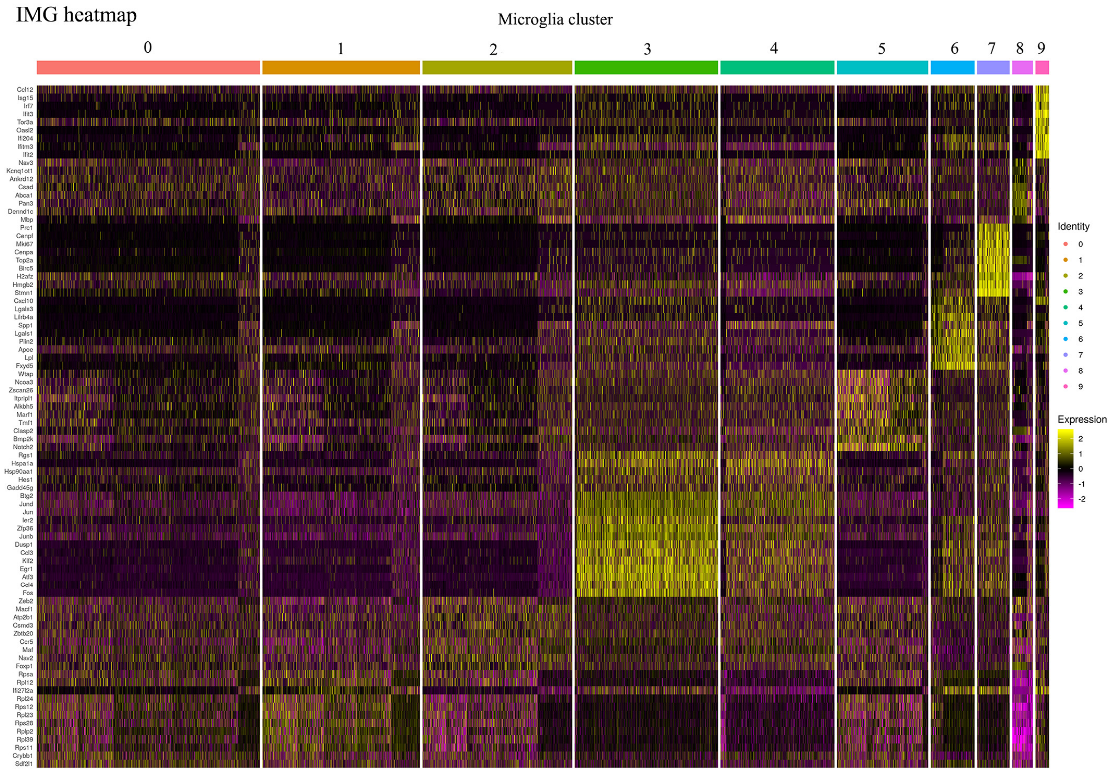


**Figure S25-** Heatmap of microglial gene expression in integration analysis. (SAH and normal microglia-IMG). IMG-C0 (*Sdf2l1* and *Crybb1),* IMG-C1 (ribosome genes), IMG-C2 (*Ccr5 and Zbtb20 etc.*) and IMG-C5 (signaling inhibitors: *Bmp2k,* transcriptional repressor: *Bhlhe41, Ncoa3*, and *Notch2 etc.*) while the other IMG clusters were predominantly from SAH microglia gene expression patterns.

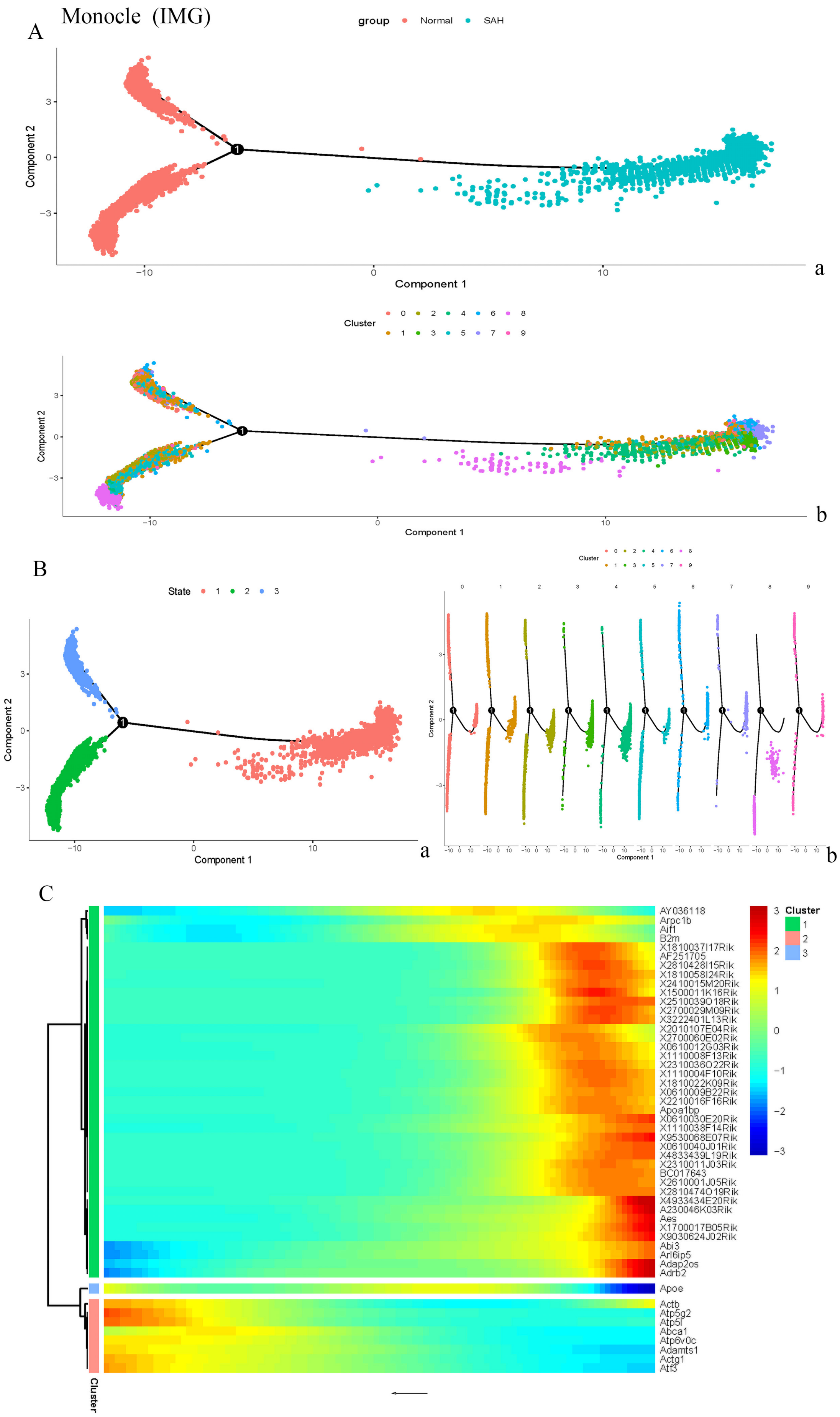
Monocle (IMG)
A
group
Normal
SAH
Component 2
Component 1
a
Cluster
b
B
State
Component 2
Component 1
a
Cluster
b
C
AY036118
Arpc1b
Aif1
B2m
X1810037I17Rik
AF251705
X2810428I15Rik
X1810058I24Rik
X2410015M20Rik
X1500011K16Rik
X2510039O18Rik
X2700029M09Rik
X3222401L13Rik
X2010107E04Rik
X2700060E02Rik
X0610012G03Rik
X1110008F13Rik
X2310036O22Rik
X1110004F10Rik
X1810022K09Rik
X0610009B22Rik
X2210016F16Rik
Apoa1bp
X0610030E20Rik
X1110038F14Rik
X9530068E07Rik
X0610040J01Rik
X4833439L19Rik
X2310011J03Rik
BC017643
X2610001J05Rik
X2810474O19Rik
X4933434E20Rik
A230046K03Rik
Aes
X1700017B05Rik
X9030624J02Rik
Abi3
Arl6ip5
Adap2os
Adrb2
Apoe
Actb
Atp5g2
Atp5l
Abca1
Atp6v0c
Adamts1
Actg1
Atf3
Cluster

**Figure S26-** Monocle analysis of IMG. **A**: Monocle- state and cluster branch; **B**: Monocle- state branch(a), monocle cluster- split branch (b) **C**: Top 50 expressed genes heatmap in monocle.
